# Unveiling Genetic Diversity, Characterization, and Selection of Bambara Groundnut (*Vigna subterranea* L. Verdc) Genotypes Reflecting Yield and Yield Components in Tropical Malaysia

**DOI:** 10.1155/2022/6794475

**Published:** 2022-04-26

**Authors:** Md Mahmudul Hasan Khan, Mohd Y. Rafii, Shairul Izan Ramlee, Mashitah Jusoh, Yusuff Oladosu, Md Al Mamun, Atiqullah Khaliqi

**Affiliations:** ^1^Laboratory of Climate-Smart Food Crop Production, Institute of Tropical Agriculture and Food Security (ITAFoS), Universiti Putra Malaysia (UPM), 43400 Serdang, Selangor, Malaysia; ^2^Bangladesh Agricultural Research Institute (BARI), Gazipur 1701, Bangladesh; ^3^Department of Crop Science, Faculty of Agriculture, Universiti Putra Malaysia (UPM), 43400 Serdang, Selangor, Malaysia

## Abstract

Addressing genetic diversity and application of appropriate breeding strategies are imperative for Bambara groundnut (*Vigna subterranea* L.) improvement as a newly introduced legume in Malaysia. It has become a “miracle lucrative” legume for Asia and Africa because of its drought resilience, excellent nutritional profiles, and versatile uses. This crop's progress has been limited owing to a lack of extensive research, marginalization, inadequate knowledge, and a lack of accessible funds, among other concerns. The expansion of this crop is reliant on the assessment and selection of unique and reliable breeding lines in various circumstances. Consequently, the goal of this work is to determine genetic diversity and the relationship between yield-contributing components in 44 Bambara groundnut accessions sourced from the Genebank of Institute of Tropical Agriculture and Food Security (ITAFoS) at Universiti Putra Malaysia (UPM). Three replications were used in the experiment, which was done using a randomized complete block design (RCBD). The data were subjected to ANOVA, PCA, correlation, and heat map cluster analysis; also, genetic parameters and broad-sense heritability estimation were carried out on recorded phenotypic descriptors. All of the investigated variables had a significant variance (*p* ≤ 0.05 or 0.01) according to the ANOVA results. Yield per hectare showed a positively strong to perfect significant correlation (0.75 ≤ *r* ≤ 1.00; *p* ≤ 0.01) with the yield components viz. fresh pod weight, hundred seed weight, dry pod weight, and dry seed weight. Interestingly, these traits had heritability ≥ 60% and genetic gain ≥ 20%, which can be beneficial for direct selection to this crop improvement. The UPGMA clustering revealed five distinct clusters in which genotypes under cluster I, cluster II, and cluster IV produce a greater yield of 5.96%, 7.12%, and 15.05%, respectively, than the grand mean yield of 1927.01 kg/ha. The PCA biplot estimated that PC1 (32.9%) and PC2 (12.9%) would cover 45.8% of the total variance. We discovered 30 promising lines that provide yields per hectare more than 1.8 ton/ha and might be used as parental lines in future breeding operations aimed at improving the grain yield in tropical areas or comparable agroecological zones.

## 1. Introduction

The Bambara groundnut (*Vigna subterranea* (L) Verde.) is a tropical legume with subterranean pods that belongs to the Fabaceae family and the Faboideae subfamily [1] and is the third most important food legume after groundnut (Arachis hypogaea L.) and cowpea (Vigna unguiculata Walp.) [2]. According to Obidiebube et al. [3], it originated in West Africa and is now a popular meal throughout Africa, able to reduce hunger, increase food safety, promote agricultural livelihoods, and assist in long-term land-use design. The world's food consumption is rising as the world's population continues to rise [3]. The existing farming method focused on intense cultivation of established crops which will be practically insufficient to provide food and nutrition security. The adoption of modern technologies is projected to play a significant role in the development of the underutilized Bambara groundnut in order to boost production and feed the world's most hungry and starving communities [4]. As an underdeveloped crop, they may have a promising future in mitigating world food demand, particularly in Africa and Asia [2]. Protein (19%), carbohydrates (63%), lipids (6.5%), and essential amino acids are all abundant in the seeds of Bambara groundnut [5]. Despite more diverse nutritional sources, breeding techniques for Bambara groundnut are not as advanced as those for peanut, chickpea, and soybean [6]. One of the reasons for Bambara groundnut's low yield is the usage of local landraces [7]. Khaliqi et al. [8] reported that the yield of Bambara groundnut varied from 0.97 ton/ha to 3.12 ton/ha, though it has the ability to produce a higher yield up to 4 to 5 ton/ha under optimum conditions and standard cultivation procedures [9, 10].

Hence, expanding research in this area may be prudent since it will provide a source of low-cost input and higher economic returns [11]. The total number of pods, dry pod weight, and hundred seed weight are essential agronomic traits since they are positively related to the overall yield [12]. The overall yield generally rises as pod weight increases and vice versa. Plant breeders require genetic resources with a high level of diversity in order to produce new varietals [13, 14]. When compared to other leguminous crops, the average yield of Bambara groundnut is considered low; nevertheless, this is mostly owing to a lack of better cultivars and modern production methods. Through a selective breeding effort, no modern high-yielding cultivars have been created throughout time. As a result, before establishing an effective breeding programme for the Bambara groundnut, a detailed study of its genetic diversity is required. As a newly introduced legume in tropical and subtropical regions, there has been little research on Bambara groundnut genetic diversity based on yield components, which contrasts with many other neglected crops [15]. Furthermore, there is relatively little evidence available on genetic variation in Bambara groundnut production and yield components [16]. The yield has been defined as a dynamic attribute controlled by polygene as well as associated with several factors that contribute to the yield [11]. Recently, some emphasis has been focused on this crop development based on the yield and its contributing characteristics [13–15, 17], with a greater degree of diversity seen in Bambara groundnut germplasms.

In crop genetic improvement for any characteristic, genetic diversity and heritability are essential considerations [15]. Additionally, since morphological traits are highly connected to the grain yield, breeders can pick which attributes to be used as a selection criterion [18]. Yield-contributing traits are mostly inherited and controlled by several environmental factors [19]. Oladosu et al. [20] found that the effect of GE interaction has virtually influenced crop growth and development. Agromorphological diversity across populations is controlled by phenological, vegetative, and yield-related characteristics [15, 17, 21]. The effectiveness of the selection is determined by the presence of a wide genetic diversity in the breeding material for the desired feature, as well as the degree to which it is heritable [22]. Knowledge of yield inheritance is essential for designing suitable breeding techniques to create better plant cultivars [23]. The best performing genotypes are chosen based on genetic diversity [24]; however, phenotypic and genotypic covariance is an indication of the nature of diversity in the breeding materials [25]. The extent of genetic susceptibility associated with some key heritable characteristics is referred to as heritability [26]. However, it has been proposed that taking heritability into account in conjunction with genetic advances provides more accurate results in a crop improvement programme [27]. In general, selecting traits with high heritability and strong genetic advance typically results in a higher yield [28]. Genetic advance describes the degree of advantage gained in a particular character under a certain selection pressure [28]. Selection based on the value of numerous genetic parameters analysis can enhance Bambara groundnut production and its related factors [15]. Bambara groundnut improvement project is required as a new crop in Malaysia to promote genetic potential and stimulate long-term research and development of new cultivars.

Considering the aforementioned incidence, we evaluated 44 Bambara groundnut accessions from selfed generation three (S3) to determine genetic inheritance and yield performance by defining genetic components, heritability, genetic advance, and ordination analysis, as well as advancing the generation as S4. Moreover, our findings will provide evidence of the genetic variation of the accessions studied, enrich the data pool, and provide an idea for better cultivation techniques for future breeding programmes by optimizing the use of limited resources and conserving biodiversity.

## 2. Materials and Methods

### 2.1. Experimental Location

The study was conducted in Ladang 15, Agricultural Research Park, Institute of Tropical Agriculture and Food Security (ITAFoS), Universiti Putra Malaysia (UPM), whose GPS location is 3°02 N latitude, 101° 42 E longitude, and 31 meters above sea level. The field experiments were conducted in Malaysia's tropical humid environment throughout the September to February cropping seasons of 2019-2020.

### 2.2. Plant Materials

Following periodic selfing and intense selection based on desirable characteristics, the seeds of chosen Bambara groundnut genotypes were kept in GenBank, ITAFoS, and UPM with proper institutional rules and guidelines. To carry out this study, seeds from 44 accessions of selfed generation three (S3) were employed.  Table 1 showcases the list of accessions from the S3 generation assessed in this investigation.

### 2.3. Experimental Design and Intercultural Practices

The soil was mechanically cultivated and harrowed prior to sowing. The seeds were planted directly into the soil at field 15 with a gap of 30 cm between plants, 50 cm between rows, 1.5 m between plots, and 2.0 m between replications using a randomized complete block design (RCBD) with three replications [17]. The experimental layout consisted of two rows of 1.6 m × 0.80 m in length. The experimental unit is made up of 10 plants per replication for each line. On a routine basis, insects, pests, and diseases were seen on the plants. Pest and disease management was carried out where it was necessary. Syngenta's Pegasus 250sc (usage at a rate of 1.0 ml/L water) was employed to control white fly, aphid, and the leaf hairy caterpillar on the field. Regular hand weeding was done as needed, and a systemic herbicide named Bayer “Roundup” (isopropylamine salt of glyphosate) was used to suppress wide leaf weeds at a concentration of 10 mL per liter of water. The research fields were irrigated on a regular basis with a sprinkler irrigation system, and weeding was controlled with silver shine covering and, if essential, manual hand weeding. NPK (15 : 15 : 15+2S) green and NPK (12 : 12 : 17-2+8S+TE) blue were applied in split doses two and six weeks after planting [8].

### 2.4. Traits Measured for Data Analysis

Twenty-seven quantifiable parameters (Table 2) were examined for phenotypic characterization based on Bambara groundnut descriptors [29]. All measurements were visually recorded in the field and postharvest lab at various development stages of five randomly selected plants based on Bambara groundnut descriptors reported by Khan et al. [17]. However, out of 27, only the 10 major (growth and yield contributing) traits are considered for presenting the results and interpretations. Moreover, for additional information, the estimated statistical results of all traits are provided in supplementary files.

### 2.5. Statistical Analysis

#### 2.5.1. Analysis of Variance (ANOVA)

Statistical analysis was done using Statistical Analysis System (SAS) version 9.4 for all of the morphological criteria specified by [29]. The means were compared at a 5% level using Duncan's New Multiple Range Test (DNMRT) method reported by Aydrous et al. [30] to differentiate the significant differences among the tested genotypes.

#### 2.5.2. Estimation of Genetic Parameters

The variance components, such as genotypic and phenotypic variance (GV and PV), were computed for each character using SAS's proc varcomp and the Restricted Maximum Likelihood (REML) approach.

- The phenotypic and genotypic coefficient of variation (PCV and GCV) was calculated as described by Singh and Chaudhary [31] as follows:

(1) $$\begin{array}{c} \text{PCV}=\frac{\surd \sigma_{\text{p}}^{2}}{\bar{X}}\times 100, \end{array}$$

(2) $$\begin{array}{c} \text{GCV}=\frac{\surd \sigma_{\text{g}}^{2}}{\bar{X}}\times 100, \end{array}$$

where *σ*_p_^2^ is the phenotypic variance, *σ*_g_^2^ is the genotypic variance, and $\bar{X}$ is the mean of the trait. GCV and PCV values were characterized as low (0-10%), moderate (10-20%), and high (20% and above) as described by Robinson and Comstock [32].

(ii) Heritability broad sense was calculated as described by Falconer and Mackay [33] which is the ratio of genetic variance (*σ*_g_^2^) to phenotypic variance (*σ*_p_^2^). The formula for broad-sense heritability is as follows:

(3) $$\begin{array}{c} h_{B}^{2}\left(\%\right)=\frac{\sigma_{\text{g}}^{2}}{\sigma_{\text{p}}^{2}}\times 100, \end{array}$$

where *σ*_g_^2^ is the genotypic variance, *σ*_p_^2^ is the phenotypic variance, and *h*_*B*_^2^ is the broad-sense heritability characterized as low (0-30%), moderate (30-60%), and high (≥60%) as given by Johnson et al. [34].

(iii) Estimated and expected genetic advance (GA). The amount of anticipated GA (as a percentage of mean) was calculated using the method outlined by Johnson et al. [34], and selection intensity (*K*) was estimated to be 5%. Following Johnson et al.'s [34] proposal, the genetic advance was classified as modest (0-10%), moderate (10-20%), and high (>20%).

(4) $$\begin{array}{c} \text{GA}\%=K\times \frac{\sqrt{\sigma_{\text{P}}^{2}}}{\bar{X}}\times h_{B}^{2}\times 100. \end{array}$$

*K* is the selection intensity (constant 5%, the value is 2.06), $\sqrt{\sigma_{\text{P}}^{2}}$ is the phenotypic standard deviation, *h*_*B*_^2^ is the heritability, and $\bar{X}$ is the mean of traits.

#### 2.5.3. Multivariate Analysis

Additionally, a dendrogram and PCA two-dimensional (2D) graph were constructed during cluster analysis based on the UPMGA (SHAN) method using NTSYSpc ver 2.0 [35] reported by Khan et al. [17]. The density plot, correlation scatter plot, correlation heat map, double dendrogram (heat map), circular plot, PCA three-dimensional (3D) graph, and contour plot were generated using NCSS 2021 (**NCSS** 2021 Statistical Software. NCSS, LLC.) reported by Khan et al. [10]. To show the graphical relationship among principal axis, eigenvalues, and cumulative variance on a single graph, “scree plot” was created using XLSTAT software (Ver.2014.5.03, Addinsoft) reported by Vidal et al. [36]. Eigen values and PCA biplot were illustrated by the use of JMP ver.16 software (SAS program) reported by Khan et al. [10]. For estimation of the Shannon diversity index, we used Multivariate Statistical Packages (MVSP) ver. 3.22 reported by Khan et al. [15]. Scatter matrix plots and genotype grouping based on the linear relationship between yield and its related component were visualized by NCSS 2021 (NCSS 2021 Statistical Software. NCSS, LLC and also reported by Khan et al. [10].

## 3. Results

### 3.1. Analysis of Variance Assessment for Quantitative Traits

Most of the plant breeders considered yield and other yield-contributing characteristics as highly important crop improvement criteria. However, in this work, we evaluated twenty-seven quantitative traits of 44 Bambara groundnut accessions to figure out how traits are inherited down over the generations and which genotypes are the best. For perfect judgments, mean square of analysis of variance for all the evaluated traits is provided in a unique table as Supplementary Table S1. Out of 27, virtually, 10 traits such as days to 50% flowering (D50%F), days to maturity (DTM), plant height (PH), total number of pods (TNP), fresh pod weight (FPW), dry pod weight (DPW), hundred seed weight (HSW), shelling percentage (SP), and harvest index (HI) are reflected as highly contributing factors to yield (Yld) per hectare, and all are important in Bambara groundnut breeding programmes.  Table 3 demonstrates the significant variation among the yields and their attributed traits as determined by analysis of variance (ANOVA).

The analysis of variance indicated that among the genotypes, there was a highly significant (*p* ≤ 0.01) difference exist in the variables. Plant height, fresh pod weight, dry pod weight, and yield per hectare exhibited highly significant (*p* ≤ 0.01) variance within the replication, whereas total number of pods, shelling percent, and harvest index indicated significant variation at the *p* ≤ 0.05 level.  Table 3 shows the minimum and maximum values for each trait across genotypes, with observed coefficients of variation (CV %) ranging from 6.35% (shelling percent) to 36.93% (biomass fresh weight). The average performance across genotypes were recoded as 38 days (D50%F), 129 days (DTM), 24.89 cm (PH), 76 (TNP), 548.49 g (FPW), 323.73 g (DPW), 331.59 g (HSW), 77.88% (SP), 57.69% (HI), and 1927.01 kg/ha for yield. Most of the attributes had a significant degree of coefficient of variation (CV %), with the highest being biomass fresh weight (36.93%), followed by biomass dry weight (27.01%) and fresh pod weight (25.02%) (Table 3).

**Figure 1:**
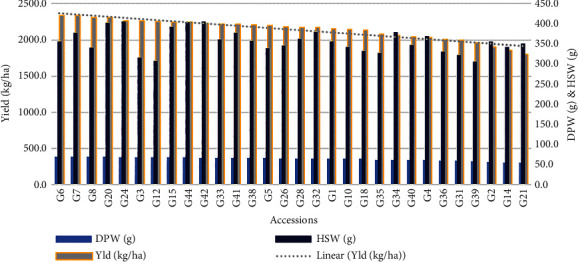
A graphical depiction of the 30 best promising genotypes based on the yield and its closely attributed parameters.

**Figure 2:**
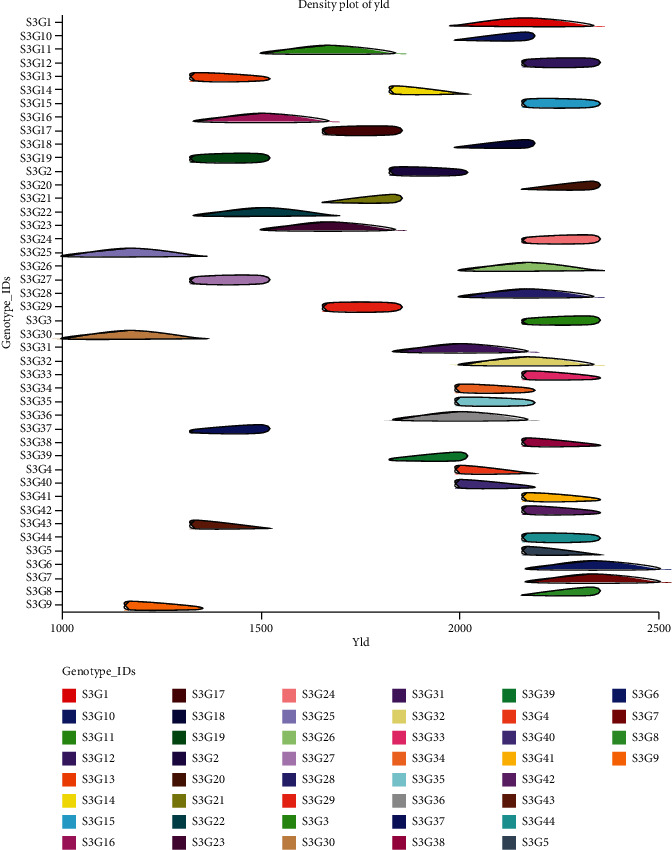
The intensity of yield potential of 44 genotypes reported by NCSS 2021 was displayed using a density map.

The mean comparison based on Duncan's New Multiple Range Test (DNMRT = 0.05) shows the performance of 44 accessions of 10 major quantitative traits (Table 4). However, for more information, the mean comparison of the rest of the traits is represented in Supplementary Table S2. We found that 77.27% of the accessions produced flowers before 40 DAS, whereas the minimum was recorded for the genotypes S3G7 (28 days) and the maximum was 50 days (S3G41). The majority (56.81%) of the accessions took less than 130 days to mature, with genotype S3G31 taking the shortest time at 117 days and genotype S3G42 taking the longest at 147 days. The maturity time of a crop is an essential factor in its improvement. The majority of plant breeders and farmers favour cultivars with high yield potential and early maturity. In our current research, we exposed the accessions S3G2 (119d), S3G5 (120d), S3G6 (117d) S3G8 (119d), S3G14 (119d), S3G16 (120d), S3G20 (120d), S3G23 (119d), S3G27 (120d), S3G28 (119d), S3G30 (120d), S3G31 (117d), and S3G34 (120d) as short duration with high yield potential. Genotype S3G38 reported the highest plant height (28.69 cm), while S3G28 recorded the lowest (20.84 cm) and the genotype S3G37 (43) had the least number of pods per plant, whereas S3G8 had the maximum (93) (Table 4). The accessions S3G6 and S3G7 had the highest dry pods per plant weights of 393.35 g and 392.43 g, respectively, whereas the genotype S3G25 and S3G30 had the lowest weight of 197.94 g. The lowest weight was recorded for the fresh pod in S3G27 (270.51 g), and the highest was 740.11 g for the genotype S3G36. The genotype S3G17 had the lowest harvest index of 43.75%, while the genotype S3G41 had the highest at 70.34%, followed by S3G26 (70.12%). The accessions of S3G9 (210.79 g) and S3G24 (406.5 g) had the lowest and maximum weight of hundred seeds among the genotypes, respectively. The shelling percentage was recorded as maximum as 70.34% (S3G41) whereas the genotype S3G13 had minimum values of 43.76%. Across the genotypes and replications, the average yield varied from 1104 to 2466 kg/ha (Table 3). However, the genotype S3G6 had the highest yield per hectare (2341.35 kg/ha), followed by the genotype S3G7 (2335.89 kg/ha), with the genotype S3G30 having the lowest yield (1178.19 kg/ha) (Table 4). The parameter yield per hectare is directly derived from the dry pod weight (g), and we found a strong association with hundred seed weight (g). Based on the yield and its highly contributing traits such as dry pod weight (DPW) and hundred seed weight (HSW), 30 best promising lines are projected in Figure 1. As shown in Figure 2, the density plot aids in understanding the general distribution of genotypes depending on the magnitude of yield potentiality.

### 3.2. Correlation Matrix Analysis

**Figure 3:**
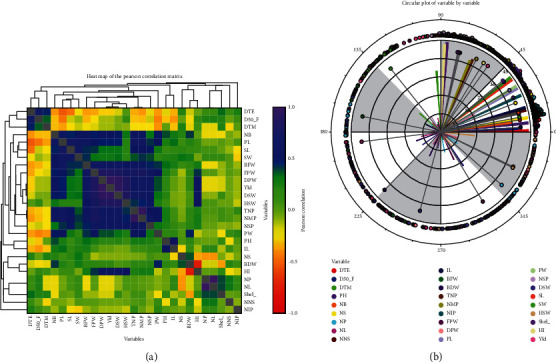
Pearson's correlation heat map (a) and circular plot (b) of 27 measurable traits revealed by NCSS 2021.

**Figure 4:**
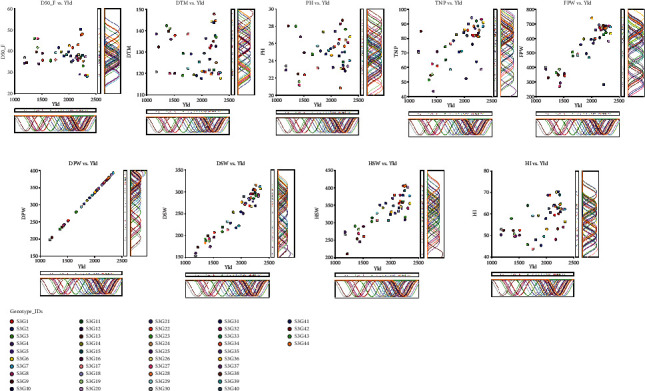
Scatter plots showing the graphical relationship between the yield and its contributing attributes revealed by NCSS 2021. On the scatter plot, a circular ring with different colors indicates the 44 accessions evaluated in this study. At the righthand side and underneath of each scatter plot, there is a bar plot and density plot which implies richness of the accession's performance for the respective traits with yield.

The phenotypic correlation of important yield-contributing traits of 44 genotypes is shown in Table 5. However, for additional understanding, the association among the twenty-seven quantitative traits is provided in Supplementary Table S3. Days to 50% percent flowering had negative and weak (0.0 ≤ *r* ≤ 0.25) as well as nonsignificant correlation with yield (*r* = −0.11; *p* ≥ 0.05). Days to maturity (*r* = −0.118) exhibited a weak (0.0 ≤ *r* ≤ 0.25), negative, and nonsignificant association with yield per hectare whereas a positive and nonsignificant correlation was reported for plant height with yield. The attribute shelling percent (*r* = −0.17) was shown to have a weak but significant (*p* ≤ 0.05) negative association with yield. A significantly positive and moderate (0.25 ≤ *r* ≤ 0.75) association was found between the trait total number of pods (*r* = 0.58; *p* ≤ 0.001) with yield. A strongly (0.75 ≤ *r* < 1.00) positive and highly significant correlation was found between the yield and its highly related traits such as fresh pod weight (*r* = 0.82; *p* ≤ 0.001), dry seed weight (*r* = 0.94; *p* ≤ 0.001), and hundred seed weight (*r* = 0.75; *p* ≤ 0.001). A correlation heat map and the circular plot were constructed based on evaluated quantitative traits (Figures 3(a) and 3(b)). The color code in the heat map indicates high (blue), low (red), and very low (yellow) association among the traits. The intensity of the hue is the indication of the magnitude of the relationship among the traits as indicated in Figure 3(a). Correspondingly, the circular plot divulged the traits' distribution based on the correlation matrix, and highly correlated traits were bunched together as shown in Figure 3(b). However, we remarked that the traits (indicating the blue hue bar) such as total number of pods, dry pod weight, fresh pod weight, hundred seed weight, and yield are highly correlated among the traits evaluated which are comparable with the result of Table 5. The scatter plots (Figure 4) illustrated the relationship between the yield and its highly contributing traits graphically: D50%F vs. yield, DTM vs. yield, PH vs. yield, TNP vs. yield, FPW vs. yield, DPW vs. yield, DSP vs. yield, HI vs. yield, and HSW vs. yield.

### 3.3. Estimation of Genetic Parameters

#### 3.3.1. Genotypic and Phenotypic Variance, Heritability, Relative Difference, and Genetic Advance

The results of the study in terms of genotypic and phenotypic variance, genotypic coefficient of variation (GCV), and phenotypic coefficient of variation (PCV), relative differences (RD), broad-sense heritability (Hb), and genetic advance (GA) as a percentage of mean are shown in Table 6. Here, we displayed and interpreted only the major traits that have a substantial contribution to yield per hectare (Table 6). However, for more clarification, the genetic parameters of all the evaluated quantitative traits are given in Supplementary Table S4. The genotypic variance (*σ*_g_^2^) ranged from 2.02 (PH) to 126876 (yield), whereas the phenotypic variance (*σ*_p_^2^) ranged from 5.70 (PH) to 130749 (yield). However, phenotypic variance appears to be greater than the genotypic variance for all traits. The fresh pod weight had greater PCV and GCV values of 25.18% and 25.07%, accordingly while shelling percent showed the lowest calculated PCV and GCV of 6.24% and 4.62%, respectively. Both the estimated PCV and GCV values were found more than 20% for the trait fresh pod weight (PCV = 25.18% and GCV = 25.07%). However, the traits including D50%F (PCV = 14.40% and GCV = 12.29%), TNP (PCV = 17.57% and GCV = 16.74%), DPW (PCV = 18.76% and GCV = 18.48%), HSW (PCV = 15.63% and GCV = 13.33%), HI (PCV = 12.87% and GCV = 12.46%), and yield (PCV = 18.76% and GCV = 18.48%) had GCV and PCV values of <20% (Table 6). This indicates that the moderate to higher variation among these traits is due to the effects of additive genes; further selection may be beneficial in improving the evaluated accessions.

*Relative difference* (RD) is an estimate of the proportion of GCV in relation to the corresponding PCV, and the calculated RD values ranged from 0.43% (fresh pod weight) to 48.15% for the plant height (Table 6). The shelling percent (26.17%) discovered a next greater difference in their PCV and GCV values compared to other remaining traits, indicating that these traits had wider genetic variability due to environmental effects and not better feedback to direct selection. Besides, traits such as days to maturity (4.72%), hundred seed weight (14.73%), fresh pod weight (0.43%), dry pod weight (1.49%), the total number of pods (4.72%), harvest index (2.46%), and yield kg/ha (1.49%) had lower relative differences (Table 6).

*Heritability* is defined as the ratio of phenotypic variation to genotypic variation for each trait. Almost all of the characteristics tested had high estimated heritability values of *h*_*b*_^2^ ≥ 30% (Table 6). Among the evaluated traits, heritability ranged from 26.88% (plant height) to 99.14% (fresh pod weight). The yield per hectare (97.04%) was the second-highest heritable trait followed by harvest index (95.15%). Generally, moderate (30% ≤ *h*_*b*_^2^ ≤ 60%) heritability values were noted for the trait shelling percent (54.51%) whereas the rest of the traits exposed high (*h*_*b*_^2^ ≥ 60%) heritability values, which indicate that the degree of traits' inheritance is less affected by the environment.

Genetic advance (GA) for plant height and fresh pod weight was recorded as a minimum (6.09%) and maximum (51.42%), respectively (Table 6). The traits, namely, days to maturity (13.73%), showed intermediate genetic advance (10% ≤ GA ≤ 20%) whereas low genetic advance (GA ≤ 10%) was recorded for traits like plant height (6.09%) and shelling percent (7.01%). The rest of the traits detected high (GA ≥ 20%) genetic advance values concurrently with high values of heritability (Table 6).

### 3.4. Assessment of the Clustering Pattern

**Figure 5:**
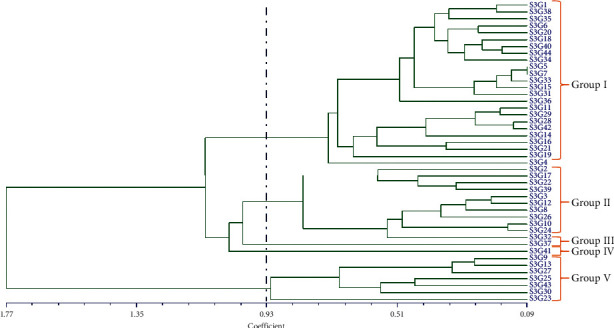
UPMGA cluster of 44 Bambara groundnut accessions based on 27 morphological features.

**Figure 6:**
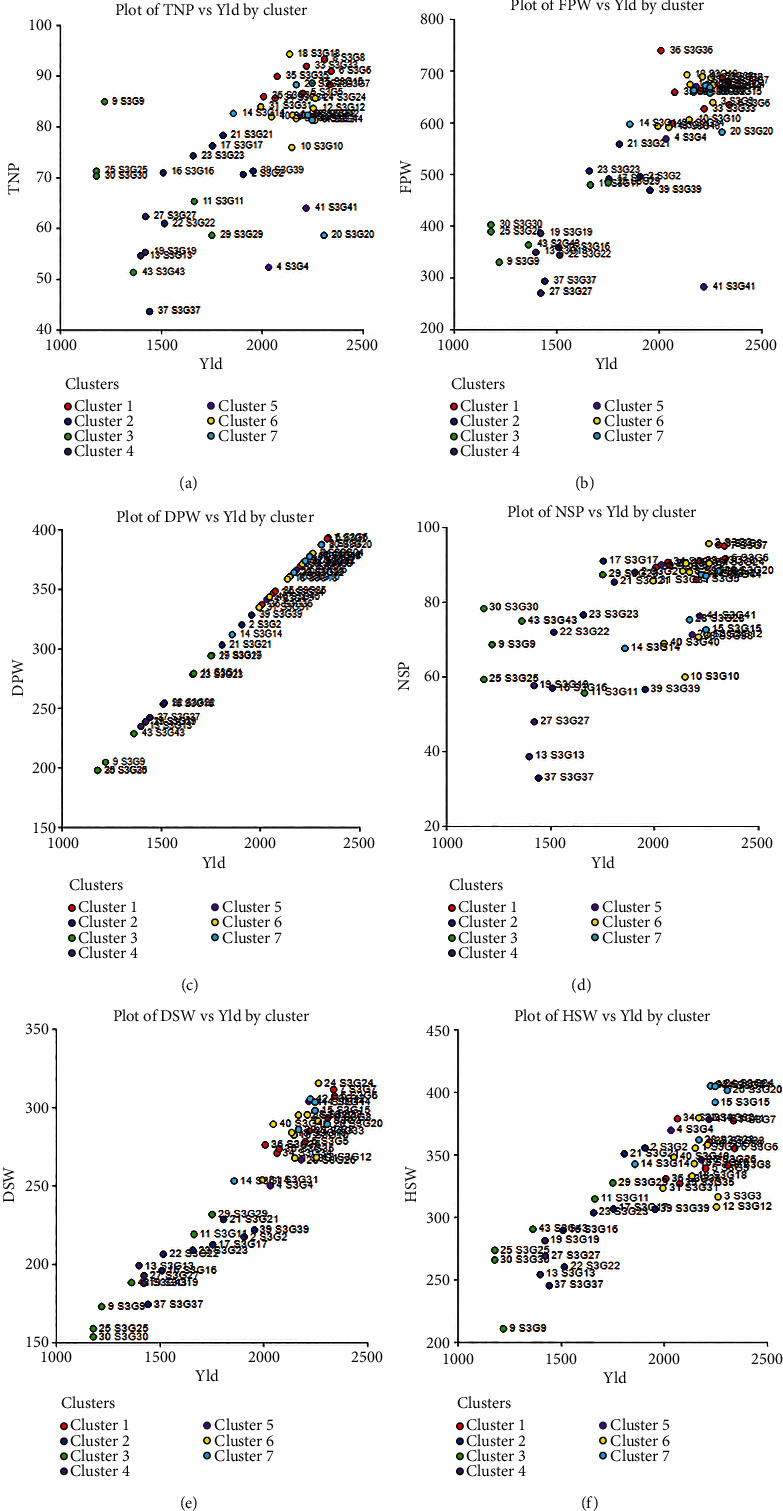
Genotype grouping and graphical visualization of the relationship between yield and its contributing components by cluster: (a) TNP vs. yield, (b) FPW vs. yield, (c) DPW vs. yield, (d) NSP vs. yield, (e) DSW vs. yield, and (f) HSW vs. yield.

Genotypic delineation is one of the key statistics in plant breeding for determining suitable genotypes for crop improvement, indicating the degree of divergence among the evaluated accessions. Clustering offers a very strong and compact illustration of the extent and pattern of genetic variation, which is crucial for selecting the desired genotype. The UPGMA (average linkage) cluster analysis revealed distinct clusters suggesting relationships among tested accessions, as represented by a dendrogram (Figure 5). As shown in Figure 5, the accessions were divided into five major clusters based on their evaluated quantifiable traits, with a dissimilarity coefficient of 0.93. The dendrogram was cut off at 0.93 using Mojena's stopping criteria to choose the best cluster number and readability. Cluster I recorded the highest number 24 (54.55%) of accessions with an average yield of 2041.95 kg/ha which was 22.41% of the grand mean yield. The maximum average yield was recorded for cluster IV (24.33%) which consists of only one accession (Table 7) followed by cluster I (24 accessions) with the best agronomic traits. Cluster II (11 accessions) assembled 25% of the accessions with an average yield of 22.66%, whereas cluster III consists of 1 accession (2.27%) with an average yield of 1441.75 kg/ha (15.82%). However, cluster V possesses 15.9% of the accessions with the lowest average yield of 1345 kg/ha. Furthermore, we observed 5.96%, 7.12%, and 15.05% greater (+) mean yield compared to the grand mean yield (1927.01 kg/ha) for cluster I, cluster II, and cluster IV, respectively, while cluster III (25.18%) and cluster V (30.20%) had lower (-) yield. On an average, in terms of yield, cluster IV (S3G41) produce 9.09% and 7.93% higher yield compared to cluster I and cluster II (Table 7). However, based on yield potentials 30 accessions including20 accessions from clusters I (ignoring S3G11, S3G29, S3G16, and S3G19), one accession from cluster IV (S3G41), and 7 accessions from cluster II (excluding S3G17 and S3G22) were identified as suitable accessions for future improvement of this crop.  Figure 6 illustrates the typical distribution of genotypes under different groups or clusters and the relationship between yield and its contributing components graphically: (a) TNP vs. yield, (b) FPW vs. yield, (c) DPW vs. yield, (d) NSP vs. yield, (e) DSW vs. yield, and (f) HSW vs. yield. In all cases, the genotypes under cluster 5, cluster 6, and cluster 7 took a similar position in the graph. The combination between dry pod weight vs. yield showed a very strong relationship which is verified by the result observed in correlation matrix analysis.

#### 3.4.1. Heat map Analysis for Genotypes and Morphological Traits

**Figure 7:**
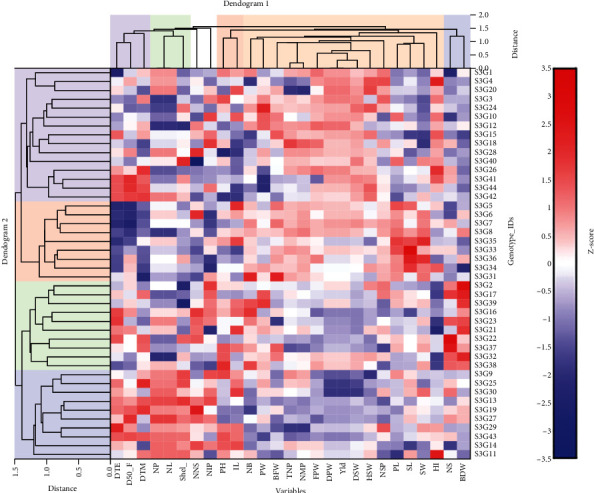
Responses to morphological descriptors of Bambara groundnut genotypes generated using NCSS 2021 as a heat map and hierarchical cluster (double dendrogram). The heat map plot describes the relative abundance of each Bambara groundnut genotype (row) within each feature (column). The color code (blue to dark red) displays the row *z*-score: red color indicates high abundance and blue color low abundance. The dendrogram shows hierarchical clustering of Bambara groundnut genotypes based on the Euclidian as the measure of distance and Ward's cluster agglomeration method.

Hierarchical clustering may be shown in two directions using the clustered heat maps (double dendrograms) technique. The heat map is a two-dimensional depiction of a data matrix, with individual cells shown as colored rectangles. A cell's color is related to its position along a color gradient. The difference in color might be seen as a difference in hue or intensity, delivering the reader with visual information about how the phenomenon of clusters are varied throughout space. It illustrates the relative patterns of high-abundance traits on a backdrop of low-abundance or nonexistent traits. Heat map analysis of morphological features based on Euclidian distance was done using the simple average clustering (SAC) approach to offer a chromatic evaluation of the Bambara groundnut genotypes. The heat map analysis produced two dendrograms: one in the vertical direction, representing traits that influenced this dispersion, and another one in the horizontal direction, representing Bambara groundnut accessions (Figure 7). Dendrogram 2 revealed four distinct clusters marked with a different color shade under two major groups as indicated in Figure 7. Cluster I covered 15 accessions followed by cluster II (10 accessions), cluster IV (10 accessions), and cluster III (9 accessions) listed in Table 8. The accessions under clusters I, II, and III perform better based on yield and its related components compared to the accession of cluster IV. This grouping pattern is verified by the result obtained from UPMGA clustering shown in Table 7, moreover, in UPMGA clustering these accessions were assembled into cluster I, cluster II, and cluster IV. Dendrogram 1 also revealed five clusters under two major groups. The maximum 15 traits were associated with cluster III while a total of 6 traits were positioned into cluster I (3) and cluster V (3) (Table 8). Total 4 traits were occupied by cluster II (2) and cluster IV (2) though, the number of nodes per stem and number of immature pods were placed into an unknown cluster (Table 8). Within each feature (column), the heat map plot depicts the relative abundance of each genotype of Bambara groundnut (row). The color coded with red represents high, whereas the color blue represents low abundance or richness. Based on the heat map plot, the traits with a red hue bar indicate high abundances to corresponded genotypes which are responsible to distinguish them from other genotypes. The genotypes such as S3G16, S3G23, and S3G29 for days to emergence; S3G44, S3G41, and S3G27 for days to 50% flowering; S3G26, S3G41, S3G44, S3G25, and S3G19 for days to maturity; S3G22, S3G37, and S3G17 for the number of stems; S3G9, S3G25, and S3G40 for shelling percent; S3G33, S3G34, and S3G36 for seed length; S3G4, S3G26, and S3G11 for harvest index; S3G31 for biomass fresh weight; and S3G2, S3G17, S3G39, S3G21, and S3G32 for biomass dry weight were detected as red to dark red color (Figure 7).

### 3.5. Assessment of Principal Component

**Figure 8:**
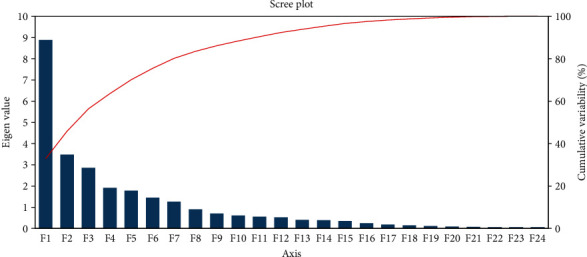
Graphical illustration of relationship among principal axis, eigenvalues, and cumulative variation revealed by XLSTAT 2014.5.

**Figure 9:**
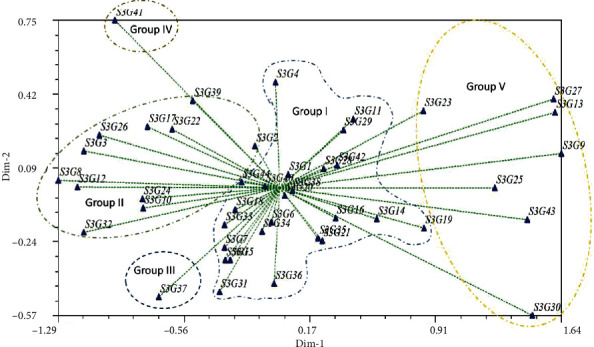
Two-dimensional (2D) elucidation of PCA discovered by NTSYSpc 2.0.

**Figure 10:**
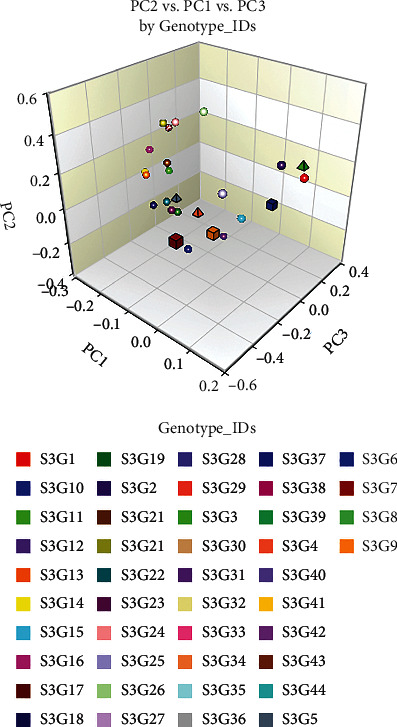
Three-dimensional (3D) elucidation of PCA discovered by NCSS 2021.

Principal component analysis (PCA) has been widely used in agricultural research for sorting traits and classifying genotypes. The first seven principal components (PC) accounted for 80.17% variation observed in the current research (Table 9). PC1 and PC2 accounted for 32.91% and 12.91% of the variance, respectively, whereas the 7th PC accounted for 4.69%. With the exception of days to emergence, days to 50% flowering, days to maturity, number of petioles and leaves, number of nodes per stem, number of immature pods, and shelling percent, the majority of the characteristics exhibited a positive correlation with PC1 (Supported data are provided in Supplementary Table S5). Fresh pods weight, dry pods weight, and yield kg per hectare showed a high coefficient (0.29), followed by 0.28 for the characteristics number of mature pods and dry seed weight. Similarly, the trait harvest index had a high and positive coefficient value (0.42) with PC2 afterward 0.30 for days to 50% flowering and 0.29 for hundred seed weight. The PC3 captured 10.60% variation and the maximum coefficient (0.54) was associated with number of petioles and leaves followed by shelling percent (0.24). The number of stems per plant had the greatest coefficient value of 0.47, followed by seed width (0.44) and associated with PC4, which contributed 7.10% of the variance. The PC5 accounted for 6.60% variation, where biomass dry weight had the highest coefficient value of 0.42, followed by number of nodes per stem (0.37) and number of immature pods (0.31). The traits days to maturity (0.24) and plant height (0.46), and number of immature pod (0.57) was strongly associated with PC6 that contributed 5.38% of the variation. Contributed variation in PC7 was 4.69%, where plant height (0.46) had the greatest coefficient value, followed by internode length (0.45) and hundred seed weight (0.25). The proportional relationship between eigenvalues and principal components, as well as their cumulative percentage of variance, is depicted in Figure 8. Taking these 7 PCs into account, it was revealed that these PCs govern the total variance (approximately 80%) for all of the assessed accessions. Moreover, the two-dimensional (2D) (Figure 9) and three-dimensional (3D) (Figure 10) graphical explication revealed that the majority of the accessions were distributed at short distances which is closer to the centroid, while the fewer were dispersed at large distances, as expressed by eigenvectors (Table 9). The genotype S3G41 was the furthest accession from the centroid in group IV; S3G37 in group III; S3G9, S3G13, S3G23, S3G27, S3G43, S3G25, and S3G30 in group V; and the remaining 35 accessions in groups I and II were assembled into near centrum as shown in Figure 9. The Shannon-Weaver diversity index was used to calculate phenotypic diversity for each feature. The calculated Shannon diversity index ranged from 1.61 to 1.64 for the characteristics examined (Table 9). The range of equitability or evenness was discovered to be between 0.98 and 1.00. The trait biomass fresh weight had the lowest diversity (*H*′ = 1.61) whereas the majority of the traits had the greatest (*H*′ = 1.64). Likewise, for almost all parameters, maximum (*E* = 1.00) evenness values were recorded, whereas biomass fresh weight per plant had the lowest (*E* = 0.98) values.

#### 3.5.1. PCA Biplot and Contour Plot Analysis

**Figure 11:**
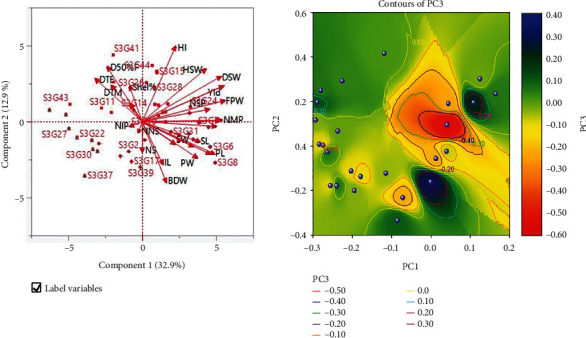
(a) PCA biplot loaded variables and accessions of Bambara groundnut. (b) Contour plot: a contour plot is a graphical approach for depicting a three-dimensional surface by displaying constant *z* (PC3) slices, known as contours, on a two-dimensional format. In other words, given a *z* (PC3) value, lines are drawn linking the (*x*, *y*) = (PC1, PC2) locations where the *z* (PC3) value occurs.

The lower angle between two vectors (Figure 11(a)) implies a higher and positive correlation (e.g., HSW versus FPW), when the angle between two vectors is less than 90°, there is no connection, and when the angle between two vectors is more than 90°, there is a negative correlation between the characteristics (e.g., HSW and NIP). PCA biplot (Figure 11(a)) loaded both variables and cases (accessions) at the same time on a single graph, shows how strongly each trait influences a principal component and correlated to each other it also shows the how distances the genotype from each other. Accessions such as S3G22, S3G27, S3G2, S3G17, S3G30, S3G37, and S3G39, as well as the characteristics number of immature pods, number of nodes per stem, and number of stems, were all positioned in the negative portion of the PCA biplot.

A contour plot can be used to show how a response variable is related to two predictor variables (Figure 11(b)). A contour plot is an alternative to a three-dimensional surface map. A contour plot is a two-dimensional representation in which all points with the same response are linked to form contour lines with constant responses. Contour plots can be used to show the relationship between two independent variables and one dependent variable. *Z* (PC3) variable values for *X* (PC1) and *Y* (PC2) variable combinations are depicted in the graph (Figure 11(b)). The PC1 and PC2 values are shown along the PC1 (*X*) and PC2 (*Y*) axes, respectively, while the PC3 value is represented by contour lines and bands. The contour lines link to PC1 and PC2 variable combinations that generate identical PC3 values. Contour plots are very useful when you need to find PC1 and PC2 combinations that result in advantageous or needed PC3 values.

## 4. Discussions

### 4.1. Analysis of Variance Assessment for Quantitative Traits

According to the results of this study, there is a significant amount of genetic diversity across Bambara groundnut accessions for the quantitative parameters evaluated. Several earlier researchers namely Khan et al. [15], Khan et al. [17], Khiliqi et al. [8], and Khaliqi [37] effectively apply a similar statistical approach in their genetic diversity research on *Vigna subterranea* (L.) that confirm our results in this tropical environment. Aliyu et al. [38] conducted a comprehensive study on Bambara groundnuts using a range of morphological features, which confirmed our research findings. In our study, the average days to 50% flowering among the 44 accessions was close to 38 days, which is lower than the (67 days) observed by Mohammed [16]. Massawe et al. [39] reported data ranging from 64 to 76 days to 50% flowering, but Masindeni [40] reported 43-80 days. Ouedraogo et al. [41] reported flowering times ranging from 32 to 53 days in Burkina Faso. Days to maturity varied substantially across the accessions, ranging from 121 to 155 days, which is comparable to the range described by Masindeni [40]. Long photoperiods promote delayed maturity of the Bambara groundnut [17]. There was a significant difference between yield and yield contributing characteristics were recorded in our study, Khan et al. [42] supported that these differences were attributable to genotype by environment (GE) effect on Bambara groundnut yield. The total number of pods, dry pod weight, and hundred seed weight in our evaluation varied from 41 to 101, 185.53 g to 414.25 g, and 198.92 g to 491.93 g, respectively. A lower trend of findings was published by Khan et al. [15] who reported 50 to 93 (TNP), 70.68 g to 359.01 g (DPW), and 177.52 to 360.15 g (HSW). It has been discovered that total number of pods, dry pod weight, and hundred seed weight is an important tool for judging genotypes which sharply connected to yield and this statement is advocated by Mohammed [16], Ntundu et al. [1], and Shegro et al. [43]. We recorded yield per hectare spanned from 1178.19 to 2341.35 kg/ha which is higher than the yield recorded by Khan et al. [15] who noted 588.98 to 2991.77 kg/ha in Malaysia as well as by Gbaguidi et al. [44] varied from 146.6 to 2678.6 kg/ha in Benin.

### 4.2. Correlation Matrix Analysis

The correlations study made a relationship between yield and the variables that contribute to it. When selecting which factors to include in the genotype selection procedure, researchers can utilize the correlation measure as a helpful tool [16]. This revealed that genotypes with a prolonged time to maturity resulted in reduced yield owing to spoilage and/or seed germination when it connected with mother plants under the soil. Our findings complemented the result reported by Mohammed [16] in Cote d'Ivoire, Gbaguidi et al. [44] in Benin among 52 landraces, and Khan et al. [17] in Malaysia. Total pods number was discovered to have a moderate and positively significant association with yield, while a positive and strong correlation with fresh dry pod weight, dry pod weight, dry seed weight, and hundred seed weight, these findings have similar trends with the results of Khan et al. [45]. Plant height and other yield-related characteristics were shown to have a positive significant correlation with field yield per hectare. Sometimes positive association of plant height may cause lower yield if the plants become bushy due to high vegetative growth though it may be a good sign when the objectives of cultivation of this crop for animal feeds as fodder. It is feasible that selecting for these characteristics may benefit both yield and fodder production in Bambara groundnut. Our conclusion is supported by previous research by Khan et al. [17], Ouedraogo et al. [41], and Onwubiko et al. [46], who found that the highly significant and positively associated characteristics are responsible to enhance the seed yield in Bambara groundnut. The combination between dry pod weight and yield showed a very strong and linear relationship because the yield per hectare was directly derived from the weight of dry pods per experimental plot. When a certain feature had an interaction with yield, the scatter diagram reflected the actual distribution of evaluated accessions. A comparable association was noted by Khan et al. [45] who found a direct and positive relationship among the investigated yield contributing traits in Bambara groundnut. However, there is no meaningful contribution on yield for traits such as number of stems, number of nodes per stem, internode length, and number of immature pods.

### 4.3. Estimation of Genetic Parameters

When the variance components such as phenotypic variance and genotypic variance were computed, it was discovered that phenotypic values were somewhat higher than the corresponding genotypic values for all traits. This implies that the trait's expression is controlled by the environment, and a previous study by Malek et al. [47] agreed with our findings. Singh and Choudhary [31] proposed a measuring scale for categorizing the genotypic coefficient of variation (GCV) and phenotypic coefficient of variation (PCV) values as low for 0 to 10%, intermediate for 10 to 20%, and high is ≥20% variance. The genotypic coefficients of variation (GCV) and phenotypic coefficients of variation (PCV) for most of the traits were medium to high, a similar pattern of the result was reported by Khan et al. [15], Khaliqi et al. [8] in Bambara groundnut diversity assessment. Direct selection would be effective using the traits of low RD values due to less influence by environmental factors. In their research, Khan et al. [17] and Umar et al. [28] found similar results, stating that the variations were present almost owing to the effect of the environment because trait improvement cannot be achieved by direct selection when high relative difference values are recorded. Past studies on heritability assessment demonstrated that selection for specific trait improvement is reliant not only on available genetic diversity but also depends on the degree of heritability [48]. Furthermore, considering heritability in couples with genetic advances gives a greater advantage than valuing heritability alone [27]. The heritability index was categorized by Johnson et al. [34] as 0 to 30% for low, 30 to 60% for intermediate, and ≥60% for high. Based on this scale most of the characteristics evaluated in this study were observed to have high heritability values, which corresponded to high genetic advances, implying a greater additive effect of genes that provide an effective selection for traits improvement directly. The findings of several investigations reported by Meena et al. [48], Langat et al. [49], and Fakuta et al. [50] offer an uninterrupted background for this conclusion. Olanrewaju et al. [51] reported genetic advance highest as 76.15% for yield and lowest 0.21% for D50%G which is higher as compared to our estimated values, this is due to the cause of variation in the genetic makeup of genotypes studied as well as the effect of environment and/or their interactions. Similarly, characteristics with low heritability and genetic advance suggested that the traits are regulated by nonadditive (dominance and/or epistasis) genes, environmental influences, or a combination of these two variables. Therefore, it is perfectly reasonable to prioritize traits with greater genotypic coefficients of variation (GCV), with the lowest relative differences, moderate to high heritability as well as genetic advance [8, 17].

### 4.4. Assessment of Clustering Pattern

Cluster analysis, a multivariate approach, was used to evaluate the genetic diversity of quantitative features by statistically grouping individuals with comparable descriptions into the same cluster. This technique is established on the distance, similarity, and relatedness of the varieties. In the distance-based strategy, there are two groups: hierarchical and nonhierarchical. Similar types are placed into one cluster based on their similarities in the hierarchical group known as “agglomerative hierarchical”. Dissimilar variations, on the other hand, are divided into various clusters. Among the several agglomerative hierarchical approaches, the unweighted pair group method with arithmetic means (UPGMA) is the most commonly employed [8]. K-means clustering is a nonhierarchical clustering approach based on a sequential threshold, parallel threshold, or optimization. This approach does not create a dendrogram or tree. In agricultural plants, the nonhierarchical clustering approach is rarely employed to examine intra-specific genetic variation [52]. In our study, we generated five unique clusters of 44 accessions of Bambara groundnut using the UPMGA method whereas a similar pattern of clustering was reported by Khaliqi et al. [8] who noted seven distinct clusters of 28 Bambara groundnut accessions. Several research, which was noted by Gbaguidi et al. [44], Gonné et al. [14], and Bonny et al. [21] also revealed significant diversity in Bambara groundnut based on morphological traits using clustering analysis. Unigwe et al. [53] utilized UPGMA model-based clustering to generate four separate groups of Bambara groundnut genotypes, whereas Atoyebi et al. [54] build a dendrogram (UPGMA) for clustering of 300 Bambara groundnut accessions. The heat map or double dendrogram is a chromatographical representation of the relationship between two components (genotypes and variables) on a unique color square bar plot. To give clear inferences we constructed a heat map and found two major groups of tested genotypes and variables. Interestingly, dendrograms 1 and 2 highlighted the groupings and sub-groups of different Bambara groundnut accessions and phenotypic characteristics. The extent of correlation among the morphological characters studied in Bambara groundnut genotypes was consistently validated using heat map analysis reported by Khan et al. [15] who grouped 15 accessions and 27 traits into two major clusters in Bambara groundnut and Virga et al. [55] also stated cluster using color-coded bar plot or heat map plot in Chilli pepper diversity study.

### 4.5. Assessment of Principal Component Analysis (PCA) and PCA Biplot

Because a few principal components initially controlled all of the information of the original variables, principal component scores were used to cluster genotypes into groups and subgroups [56]. The principal component analysis is the most frequently utilized re-justification approach for cluster analysis. Khan et al. [17] classified genetically identical accessions as belonging to the same group, but genetically diverse individuals might exhibit a wide range of heterosis. The objective of the principal component analysis is to determine the total variance in a collection of variables that account for the most variability in the data in a sequential manner [17]. In general, traits are inter-correlated to various degrees, thus all of the principal components are not necessary to properly summarise the data. The first axis (PC1) of any principal component analysis (PCA) explains the highest amount of the overall variation [15]. Shegro et al. [43] classified the 20 Bambara groundnut accessions based on quantitative features using PCA analysis. In our findings, the percentages of variation for PC1 and PC2 was 32.91% and 12.91%, accordingly, whereas Mohammed et al. [57] discovered that PC1 and PC2 contributed 19% and 14%, respectively, and Olanrewaju et al. [51] reported to 24.67% for PC1 and 17.63% for PC2 in Bambara groundnut. Mustafa et al. [58] observed similar results and concluded that accessions diversity was governed by the characteristics with higher eigenvalues. The observed diversity index value for the majority of the features examined in our study was *H*′ ≥ 1.63, which is supported by Aliyu et al. [38], who reported *H*′ index values ranging from 1.60 to 2.07, whereas Olukolu et al. [2], reported 0.1 to 0.15 for 19 qualitative and 0.09 to 0.16 for 28 quantitative traits of 124 Bambara groundnut accessions. A standard value for *H*′ Index is 1.5 to 3.5, reported by Khan et al. [15]. However, based on this scale the estimated *H*′ index values for evaluated traits in our study showed moderate genetic diversity and this is due to the cleistogamous type of reproductive system in Bambara groundnut [15]. As a self-pollinated crop, Bambara groundnut diversity is influenced by farmers' agricultural practices as well as seed management strategies such as recycling, storing, trading, and introducing new species, according to the findings of Khan et al. [17]. PCA biplot showed that certain accessions and characteristics could be less useful for crop development. Similar findings have been reported by Khan et al. [15] and Alkan et al. [59]. The contour lines and bands make it straightforward to find combinations that produce the necessary values. Madamba [60] successfully uses a contour plot for crop selection; moreover, Wnuk et al. [61] use a contour plot for crop harvest index visualization.

## 5. Conclusion

The current study revealed significant levels of diversity across the accessions as well as yield and its contributing components in terms of all statistical parameters. It is evident that the boosting yield and other yield-related attributes may be achieved by efficient selection based on heritability and genetic advance estimations and recommended that direct selection based on these traits will be beneficial for crop improvement. The first two main components accounted for 45.8% of the total variance, and owing to persistent selfing, the genotypes become homozygous and had a moderate degree of divergence as measured by the *H*-index (1.63 to 1.64). Clustering and heat map analysis revealed that the majority of the high-producing accessions were grouped together in the same cluster, i.e., cluster I, based on the Euclidian distance. Moreover, 20 accessions from cluster I, 9 accessions from cluster II, and 1 accession from cluster IV were identified as high-performing accessions and can be recommended as high-yield potentials. We recommend that seeds from the S3 generation can be grown for further confirmation as well as advancing the generation and selection of the best accessions from the mega environmental trials along with molecular characterizations. However, our findings will provide evidence on the genetic variation of the accessions studied and expand the data pool for the upcoming breeding program through optimum uses of limited genetic materials.

## Figures and Tables

**Table 1:** List of Bambara groundnut accessions used in this current study.

Selfed generation S3					
Accessions	Code	Accessions	Code	Accessions	Code
DunP2-18	S3G1	BdilaP10-18	S3G17	GiiwP12-18	S3G33
DunP8-18	S3G2	BdilaP8-18	S3G18	GiiwP11-18	S3G34
DunP9-18	S3G3	BdilaP11-18	S3G19	GiiwP9-18	S3G35
DunP6-18	S3G4	BdilaP5-18	S3G20	GiiwP1-18	S3G36
MaikP11-18	S3G5	JataP3-18	S3G21	KarP3-18	S3G37
Maik12-18	S3G6	JataP5-18	S3G22	KarP10-18	S3G38
MaikP3-18	S3G7	JataP4-18	S3G23	KarP9-18	S3G39
MaikP6-18	S3G8	JataP1-18	S3G24	KarP8-18	S3G40
CancP1-18	S3G9	MaibP3-18	S3G25	ExSokP4-18	S3G41
CancP2-18	S3G10	MaibP8-18	S3G26	ExSokP3-18	S3G42
CancP4-18	S3G11	MaibP9-18	S3G27	ExSokP10-18	S3G43
CancP3-18	S3G12	MaibP6-18	S3G28	ExSokP5-18	S3G44
RokP6-18	S3G13	KataP4-18	S3G29		
RokP9-18	S3G14	KataP1-18	S3G30		
RokP1-18	S3G15	KataP5-18	S3G31		
RokP3-18	S3G16	KataP8-18	S3G32		

**Table 2:** List of twenty-seven quantitative traits and their measuring procedures.

Sl. no	Quantitative traits	Code	Procedure of assessment
1	Days to emergence	DTE (d)	The length of time required from planting to the first typical leaf appearing on the soil surface
2	Days to 50% flowering	D50%F (d)	The time frame between seed germination to the appearance of 50% flowering
3	Days to maturity	DTM (d)	From sowing till the first day of harvest, the days are counted
4	Plant height	PH (cm)	Measured from the soil surface level to the tip of the topmost terminal leaflet of 10-week aged plants
5	No. of branches/stem	NB	Data were collected from five stems of five healthy plants at the time of harvest
6	No. of stems/plant	NS	Data were collected from five healthy plants at the time of harvest
7	No. of petioles/plant	NP	After two weeks of the first flowering, data was taken at random from five healthy plants
8	No. of leaves/plant	NL	After two weeks of the first flowering, data was taken at random from five healthy plants
9	No. of nodes per stem	NNS	Data were collected from five stems of five healthy plants at the time of harvest
10	Internode length	IL (cm)	The average length of the 4th internode was randomly selected from the five longest stems of five healthy plants after ten weeks of seeding
11	Biomass fresh weight/plant	BFW (g)	At harvesting, a random average of 5 fresh plants was counted
12	Biomass dry weight/plant	BDW (g)	The weight of the dried plant was measured after the harvested plant was dried in the sun
13	Total no. of pods/plant	TNP	During harvesting, the data were counted, and the average values from five plants were chosen at random
14	No. of mature pods	NMP	During harvesting, the data were counted, and the average values from five plants were chosen at random
15	No. of immature pods/plant	NIP	During harvesting, the data were counted, and the average values from five plants were chosen at random
16	Fresh pod weight	FPW (g)	Using an OHAUS Precision Standard Measuring Scale, randomly average values from 5 plants were recorded at the time of harvest
17	Dry pod weight	DPW (g)	Fresh pods were sun-dried up to 12% moisture; then, data was counted
18	Pod length	PL (mm)	Data were collected within two months of harvest and were averaged from five pods at random. A digital Vernier caliper (cat. no. 14-648-17, Fisher Brand Traceable, China) was used to measure the pod length
19	Pod width	PW (mm)	Data were collected within two months of harvest and were averaged from five pods at random. A digital Vernier caliper (cat. no. 14-648-17, Fisher Brand Traceable, China) was used to measure the pod width
20	No. of seeds/plant	NSP	After dehusking all pods, data were recorded; randomly average values from 5 plants were used
21	Dry seed weight/plant	DSW (g)	Within two months of harvest, data was collected after drying seeds (12% moisture) on an OHAUS Precision Standard Measuring Scale
22	Seed length	SL (mm)	Data were collected within two months of harvest and were averaged from five pods at random. A digital Vernier caliper (cat. no. 14-648-17, Fisher Brand Traceable, China) was used to measure the seed length
23	Seed width	SW (mm)	Data were collected within two months of harvest and were averaged from five pods at random. A digital Vernier caliper (cat. no. 14-648-17, Fisher Brand Traceable, China) was used to measure the seed width
24	100 seed weight	HSW (g)	Within two months of harvest, hundred seed weight was collected on an OHAUS Precision Standard Measuring Scale
25	Shelling percentage	SP (%)	Within two months of harvest, the ratio of dry seed weight and dry pod weight was determined (at 12% moisture content)
26	Harvest index	HI (%)	Grain yield (kg per ha)/biological yield (grain + straw) × 100 is calculated to measure the harvest index (%)
27	Yield kg per hectare	Yld (kg/ha)	The plot yield was then converted to a kilogramme per hectare (kg/ha) using data weight of dried pods (at 12% moisture content) per plot

**Table 3:** Mean square and coefficient of variance estimation for yield and its attributed traits revealed by ANOVA.

Trait	Replication (df = 2)	Genotype (df = 43)	Mean ± SE	Max.	Min.	CV (%)
D50%F	5.55	73.17^∗∗^	37.91 ± 0.47	52.00	26.00	14.31
DTM	12.19	252.72^∗∗^	129 ± 0.82	149.00	116.00	7.30
PH	35.8^∗∗^	11.53^∗∗^	24.89 ± 0.24	31.37	15.31	11.31
TNP	103.05^∗^	503.18^∗∗^	76.08 ± 1.16	101.00	41.00	17.51
FPW	3358.21^∗∗^	56883.56^∗∗^	548.49 ± 11.94	753.05	258.97	25.02
DPW	2940.74^∗∗^	10852.22^∗∗^	323.73 ± 5.28	414.25	185.53	18.74
HSW	398.97	6592.91^∗∗^	331.59 ± 4.48	491.93	198.92	15.53
SP	78.71^∗^	49.34^∗∗^	77.88 ± 0.43	86.29	63.69	6.35
HI	18.27^∗^	160.18^∗∗^	57.69 ± 0.64	70.80	42.72	12.81
Yld	104193.89^∗∗^	384501.04^∗∗^	1927.01 ± 31.42	2466.00	1104.00	18.74

“∗∗” is significant at the 0.01 level; “∗” is significant at the 0.05 level. df = degree of freedom; max = maximum (across genotypes); min = minimum (across genotypes); CV = coefficient of variation; D50%F = days to 50% flowering (d); DTM = days to maturity (d); PH = plant height (cm); TNP = total number of pods; FPW = fresh pod weight (g); DPW = dry pod weight (g); HSW = hundred seed weight (g); SP = shelling percent; HI = harvest index (%); Yld = yield (kg/ha).

**Table 4:** Mean performance and their comparison of ten major phenotypic traits among the evaluated 44 accessions.

Genotypes	D50%F	DTM	PH	TNP	FPW	DPW	HSW	SP	HI	Yld
S3G1	37f-j	132ij	26.48a-e	82.33e-j	674.29b-e	361.21d-g	355.4d-h	74.22m-q	59.45f-h	2150.08d-g
S3G2	37.67e-i	119o	24.77b-h	70.67lm	496.21lm	320.10k-m	355.55d-h	68.13rs	45.41st	1905.4k-m
S3G3	33jk	137e-h	27.62a-c	81.33g-j	639.67fg	379.85a-c	316.39h-n	76.77f-p	64.69b	2260.99a-c
S3G4	37.67e-i	121m-o	23.21d-i	52.33rs	568.87jk	341.39ij	369.68a-f	73.42m-r	68.79a	2032.06ij
S3G5	29kl	120.33	24.98a-h	86.67b-g	662.11de	369.65c-e	339.08e-k	75.26j-q	62.40b-e	2200.3c-e
S3G6	28.67kl	117.67o	26.77a-d	91abc	635.22g	393.35a	355.16d-h	78.26d-m	56.38ij	2341.35a
S3G7	28l	120.33no	24.00c-i	88.33a-f	680.43b-d	392.43a	377.23a-e	79.44b-l	62.16b-e	2335.89a
S3G8	28.67kl	119.67o	26.13a-f	93.33a	686.92bc	387.77ab	341.58e-k	75.68i-q	53.86jk	2308.15ab
S3G9	34.67g-j	132.33h-j	28.04ab	85c-g	330.15q	204.74r	210.79s	84.49ab	52.40k-n	1218.69r
S3G10	37.67e-i	126.67kl	24.55b-i	76j-l	606.01h	360.48e-h	342.84e-k	78.37d-m	64.13b	2145.7e-h
S3G11	35.33f-j	129.33jk	24.7b-h	65.33m-o	479.97mn	279.45o	314.66h-n	78.50d-m	63.98b	1663.35o
S3G12	38e-i	132.67h-j	22.82e-i	83.67d-h	687.17bc	378.54a-c	308.28i-o	70.92q-s	60.977c-f	2253.22a-c
S3G13	39.67c-f	139.67c-f	22.47f-i	54.67q-s	349.37pq	234.85q	254.21q-s	84.86a	52.22k-o	1397.9q
S3G14	39.67c-f	119.33o	27.35a-c	82.67e-i	597.42hi	311.95lm	342.51e-k	81.09a-h	59.25f-h	1856.85lm
S3G15	38e-i	137.67d-g	25.03a-g	88.67a-e	657.79ef	377.51a-d	392.13a-d	79.04c-l	68.93a	2247.07a-d
S3G16	39c-h	120o	24.94a-h	71lm	358.56p	253.46p	289.58l-q	77.48e-o	50.07n-q	1508.71p
S3G17	36f-j	138.33d-f	23.14d-i	76.33i-l	491.54lm	294.64no	306.68j-o	72.19o-s	43.757t	1753.83no
S3G18	36.67f-j	121.67m-o	25.99a-f	94.33a	693b	358.66e-h	333.07f-l	79.23b-l	63.77b	2134.86e-h
S3G19	41.67b-e	141b-e	21.58g-i	55.33q-s	386.20o	238.74pq	281.21m-r	78.87c-l	50.64l-p	1421.09pq
S3G20	37.33e-j	120.33no	23.35d-i	58.67p-r	582.11ij	387.41ab	401.66a-c	74.69k-q	62.34b-e	2306.01ab
S3G21	41.67b-e	129jk	25.14a-g	78.33h-k	558.55k	303.29mn	350.9d-i	75.40i-q	47.97q-s	1805.27mn
S3G22	35.67f-j	137.67d-g	22.37f-i	61o-q	343.76pq	254.49p	260.42p-r	81.21a-h	52.42k-n	1514.84p
S3G23	35.67f-j	119o	24.79b-h	74.33kl	506.92l	278.49o	303.7k-p	75.14j-q	45.83r-t	1657.68o
S3G24	35.33f-j	125k-n	26.41a-e	85.67b-g	672.81b-e	380.18a-c	406.5a	83.04a-d	62.40b-e	2262.99a-c
S3G25	34.33ij	138.33d-f	22.92e-i	71.33lm	389.35o	197.96r	273.64n-r	80.27a-j	50.32m-q	1178.32r
S3G26	38.33d-i	144.33a-c	23.07d-i	82f-j	669.97c-e	366.30c-e	345.99e-k	72.87n-r	70.12a	2180.38c-e
S3G27	46ab	120o	21.18hi	62.33op	270.51s	238.92pq	269.04o-r	80.63a-i	49.63o-q	1422.19pq
S3G28	43bc	119.67o	20.84i	88.33a-f	662.71de	364.23c-f	362.02b-g	78.59d-m	63.39bc	2168.04c-f
S3G29	39.33c-g	122.33l-o	25.72a-f	58.67p-r	484.09mn	294.14no	327.57f-l	78.72d-m	59.23f-h	1750.83no
S3G30	37f-j	120.67m-o	23.40d-i	70.33l-n	402.79o	197.94r	265.91o-r	77.88d-n	52.83k-m	1178.19r
S3G31	39.67c-f	117.67o	25.42a-f	84d-h	593.62hi	334.76i-k	323.32g-m	75.93h-q	57.83hi	1992.62i-k
S3G32	35.67f-j	133g-j	28.27ab	81.67g-j	659.54ef	364.15c-f	379.56a-e	81.16a-h	48.187p-r	2167.55c-f
S3G33	37f-j	121.67m-o	23.86c-i	92ab	627.47g	372.76b-e	360.39c-g	76.60g-p	62.9b-d	2218.84b-e
S3G34	41.67b-e	120.67m-o	25.65a-f	85.67b-g	598.54hi	346.74g-i	378.89a-e	78.13d-n	68.65a	2063.9g-i
S3G35	35.67f-j	136.33e-i	27.42a-c	90a-d	658.94ef	348.52f-i	327.05f-l	78.77d-l	60.66d-g	2074.54f-i
S3G36	42.67b-d	121m-o	24.65b-h	86b-g	740.11a	337.18ij	330.94f-l	81.99a-f	52.90k-m	2007ij
S3G37	34ij	136f-i	26.77a-d	43.67t	293.63r	242.21pq	245.22rs	72.14p-s	49.70o-q	1441.75pq
S3G38	36.67f-j	135f-i	28.69a	82.33e-j	689.37bc	371.35b-e	358.03c-h	79.58a-k	53.147kl	2210.45b-e
S3G39	38.33d-i	125.33k-m	25.2a-g	71.33lm	469.32n	328.41j-l	306.46j-o	67.50s	49.55pq	1954.82j-l
S3G40	35g-j	129.33jk	25.57a-f	82f-j	591.34hi	343.69h-j	348.03e-j	84.17a-c	62.61b-e	2045.77h-j
S3G41	50.33a	143.67a-c	23.46d-i	64n-p	282.61rs	372.46b-e	378.31a-e	81.7a-g	70.34a	2217.02b-e
S3G42	47.67a	147.67a	23.12d-i	82.33e-j	672.03c-e	373.79b-e	405.2ab	81.80a-g	59.97e-h	2224.91b-e
S3G43	46ab	142.33b-d	28ab	51.33s	363.71p	228.82q	290.49l-q	82.35a-e	58.01g-i	1362.01q
S3G44	48a	144.67ab	25.68a-f	81.33g-j	668.77c-e	377.51a-d	404.78ab	80.46a-j	64.403b	2247.07a-d
Mean	37.91 ± 0.47	129.01 ± 0.82	24.90 ± 0.25	76.08 ± 1.16	548.48 ± 11.94	323.73 ± 5.28	331.59 ± 4.48	77.88 ± 0.43	57.69 ± 0.64	1927.01 ± 31.42
St. dev	5.42	9.41	2.82	13.33	137.23	60.65	51.49	4.94	7.39	361.03

St. dev. = standard deviation; D50%F = days to 50% flowering (d); DTM = days to maturity (d); PH = plant height (cm); TNP = total number of pods; FPW = fresh pod weight (g); DPW = dry pod weight (g); HSW = Hundred seed weight (g); SP = Shelling percent; HI = harvest index (%);Yld = yield (kg/ha). The genotypes with the same letter in the same column are statistically similar at DNMRT = 0.05.

**Table 5:** Estimation of Pearson's correlation matrix for major yield-contributing traits of 44 Bambara groundnut accessions.

Traits	D50%F	DTM	PH	TNP	FPW	DPW	DSW	HSW	SP	HI	Yld
D50%F	1	0.296^∗^	-0.194^∗^	-0.239^∗^	-0.214^∗^	-0.11	-0.047	0.133	0.178^∗^	0.104	-0.11
DTM		1	-0.031	-0.228^∗^	-0.183^∗^	-0.118	-0.058	-0.099	0.190^∗^	0.076	-0.118
PH			1	0.171^∗^	0.166	0.095	0.114	0.038	0.045	-0.019	0.095
NB				0.528^∗∗^	0.429^∗∗^	0.481^∗∗^	0.434^∗∗^	0.289^∗^	-0.166^∗^	-0.002	0.481^∗∗^
TNP				1	0.732^∗∗^	0.584^∗∗^	0.594^∗∗^	0.379^∗∗^	0.037	0.241^∗^	0.584^∗∗^
FPW					1	0.815^∗∗^	0.784^∗∗^	0.638^∗∗^	-0.1	0.411^∗∗^	0.815^∗∗^
DPW						1	0.943^∗∗^	0.752^∗∗^	-0.179^∗^	0.569^∗∗^	1.00^∗∗^
DSW							1	0.782^∗∗^	0.15	0.598^∗∗^	0.943^∗∗^
HSW								1	0.045	0.482^∗∗^	0.752^∗∗^
SP									1	0.103	-0.179^∗^
HI										1	0.569^∗∗^
Yld											1

“∗∗” indicates that the correlation is significant at the 0.01 level; “∗” indicates that the correlation is significant at the 0.05 level. D50%F = days to 50% flowering (d); DTM = days to maturity (d); PH = plant height (cm); TNP = total number of pods; FPW = fresh pod weight (g); DPW = dry pod weight (g); DSW = dry seed weight (g); HSW = hundred seed weight (g); SP = shelling percent; HI = harvest index (%); Yld = yield (kg/ha).

**Table 6:** Estimation of genetic parameters for major yield-contributing traits of 44 Bambara groundnut accessions.

Traits	Mean	*σ*_e_^2^	*σ*_g_^2^	*σ*_p_^2^	PCV (%)	GCV (%)	RD (%)	*h*_*b*_^2^ %	GA (%)
D50%F	37.91	8.09	21.69	29.78	14.40	12.29	14.65	72.84	21.60
DTM	129.01	8.27	81.48	89.76	7.34	7.00	4.72	90.78	13.73
PH	24.90	5.48	2.02	7.50	11.00	5.70	48.15	26.88	6.09
TNP	76.08	16.48	162.23	178.71	17.57	16.74	4.72	90.78	32.86
FPW	548.49	164.37	18906.40	19070.77	25.18	25.07	0.43	99.14	51.42
DPW	323.74	109.31	3581.00	3690.31	18.76	18.48	1.49	97.04	37.51
HSW	331.59	733.20	1953.20	2686.40	15.63	13.33	14.73	72.71	23.41
SP	77.89	10.74	12.87	23.60	6.24	4.61	26.17	54.51	7.01
HI	57.70	2.68	52.50	55.18	12.87	12.56	2.46	95.15	25.23
Yld	1927.01	3873.00	126876.00	130749.00	18.76	18.48	1.49	97.04	37.51

*σ*_*e*_^2^ = error variance; *σ*_g_^2^ = genotypic variance; *σ*_p_^2^ = phenotypic variance;  *h*_*b*_^2^ = heritability in broad sense; PCV = phenotypic coefficient of variation; GCV = genotypic coefficient of variation; RD = relative difference; GA = genetic advance; D50%F = days to 50% flowering (d); DTM = days to maturity (d); PH = plant height (cm); TNP = total number of pods; FPW = fresh pod weight (g); DPW = dry pod weight (g); DSW = dry seed weight (g); HSW = hundred seed weight (g); SP = shelling percent; HI = harvest index (%); Yld = yield (kg/ha).

**Table 7:** Relative proportion of grand mean yield for five clusters based on the UPMGA clustering pattern.

Cluster	Accession number	Accessions	Average yield (kg/ha)	RPGY (%)
I	24 (54.55%)	S3G1, S3G5, S3G6, S3G7, S3G18, S3G31, S3G33, S3G34, S3G35, S3G36, S3G40, S3G42, S3G44, S3G20, S3G15, S3G4, S3G11, S3G29, S3G14, S3G28, S3G38, S3G16, S3G19, and S3G21	2041.95 (22.41%)	(+) 5.96
II	11 (25%)	S3G3, S3G8, S3G10, S3G12, S3G24, S3G26, S3G32, S3G2, S3G17, S3G39, and S3G22	2064.35 (22.66%)	(+) 7.12
III	1 (2.27%)	S3G37	1441.75 (15.82%)	(-) 25.18
IV	1 (2.27%)	S3G41	2217.02 (24.33%)	(+) 15.05
V	7 (15.9%)	S3G9, S3G13, S3G23, S3G27, S3G25, S3G30, and S3G43	13.45 (14.76%)	(-) 30.20

Grand average yield = 1927.01 kg/ha; relative proportion of grand average yield = RPGY (%); “(+)” = yield higher; “(-)” = yield lower.

**Table 8:** Genotype and variable differentiation based on the double dendrogram.

Cluster	Genotypes
1	S3G1, S3G3, S3G4, S3G10, S3G12, S3G15, S3G18, S3G20, S3G24, S3G26, S3G28, S3G40, S3G41, S3G42, and S3G44
2	S3G2, S3G16, S3G17, S3G21, S3G22, S3G23, S3G32, S3G37, S3G38, and S3G39
3	S3G5, S3G6, S3G7, S3G8, S3G31, S3G33, S3G34, S3G35, and S3G36
4	S3G9, S3G11, S3G13, S3G14, S3G19, S3G25, S3G27, S3G29, S3G30, and S3G43
Cluster	Variables
1	DTE, D50%F, and DTM
2	PH, IL
3	NB, BFW, TNP, NMP, FPW, DPW, PL, PW, NSP, DSW, SL, SW, HSW, HI, and Yld
4	NS, BDW
5	NP, NL, and Shel%
None	NNS, NIP

**Table 9:** Estimation of Shannon's diversity index (*H*′) and principal component analysis (PCA) of 44 Bambara groundnut accessions.

Parameters	PC1	PC2	PC3	PC4	PC5	PC6	PC7	
Eigenvalue	8.88	3.49	2.86	1.92	1.78	1.45	1.27	
Proportion of variance (%)	32.91	12.91	10.6	7.1	6.6	5.38	4.69	
Cumulative variance (%)	32.91	45.82	56.41	63.51	70.1	75.48	80.17	
Shannon's diversity index (*H*′)								
Trait	*H*′ index	Evenness (*E*)	Traits	*H*′ index	Evenness (*E*)	Traits	*H*′ index	Evenness (*E*)
DTE	1.63	0.99	IL	1.64	1	PW	1.64	1
D50%F	1.64	1	BFW	1.61	0.98	NSP	1.63	0.99
DTM	1.64	1	BDW	1.63	0.99	DSW	1.64	1
PH	1.64	1	TNP	1.64	1	SL	1.64	1
NB	1.64	1	NMP	1.63	0.99	SW	1.64	1
NS	1.64	1	NIP	1.64	1	HSW	1.64	1
NP	1.63	0.99	FPW	1.63	0.99	SP	1.64	1
NL	1.63	0.99	DPW	1.64	1	HI	1.64	1
NNS	1.64	1	PL	1.64	1	Yld	1.64	1

DTE = days to emergence (d); D50%F = days to 50% flowering (d); DTM = days to maturity (d); PH = plant height (cm); NB = number of branches per plant; NS = number of stems per plant; NP = number of petioles per plant; NL = number of leaves per plant; NNS = no. of nodes per stem; IL = internode length (cm); BFW = biomass fresh weight per plant (g); BDW = biomass dry weight per plant (g); TNP = total no. of pods per plant; MP = number of mature pods per plant; IMP = number of immature pods per plant; FPW = fresh pod weight (g); DPW = dry pod weight (g); PL = pod length (mm); PW = pod width (mm); NSP = number of seeds per plant; DSW = dry seed weight per plant (g); SL = seed length (mm); SW = seed width (mm); HSW = hundred seed weight (g); SP = shelling percent; HI = harvest index (%); Yld = yield (kg/ha).

## Data Availability

All data are provided in Results of this manuscript.

## Supplementary Materials

Supplementary Materials Table S1: mean square and coefficient of variance estimation for quantitative traits revealed by ANOVA. Table S2: mean performance and comparison of 44 accessions based on 27 phenotypic traits revealed by ANOVA. Table S3: estimation of Pearson's correlation matrix for 27 quantitative traits of 44 Bambara groundnut accessions. Table S4: estimation of genetic parameters for 27 quantitative traits of 44 Bambara groundnut accessions. Table S5: estimation of Shannon's diversity index (*H*′) and principal component analysis (PCA) of 44 Bambara groundnut accessions.
