# Supplementary material for: Unveiling Genetic Diversity, Characterization, and Selection of Bambara Groundnut (Vigna subterranea L. Verdc) Genotypes Reflecting Yield and Yield Components in Tropical Malaysia
Source: Biomed Res Int. 2022 Apr 26;2022:6794475. doi: 10.1155/2022/6794475 (PMC9071916; doi:10.1155/2022/6794475)
Supplement: Supplementary Materials — Table S1: mean square and coefficient of variance estimation for quantitative traits revealed by ANOVA. Table S2: mean performance and comparison of 44 accessions based on 27 phenotypic traits revealed by ANOVA. Table S3: estimation of Pearson's correlation matrix for 27 quantitative traits of 44 Bambara groundnut accessions. Table S4: estimation of genetic parameters for 27 quantitative traits of 44 Bambara groundnut accessions. Table S5: estimation of Shannon's diversity index (H′) and principal component analysis (PCA) of 44 Bambara groundnut accessions. [file 6794475.f1.pdf]

Unveiling Genetic Diversity, Characterization and Selection of Bambara groundnut (*Vigna subterranea* L. Verdc) Genotypes Reflecting Yield & Yield Components in Tropical Malaysia

Md Mahmudul Hasan Khan[1, 3*], Mohd Y. Rafii[1, 2*], Shairul Izan Ramlee[2], Mashitah Jusoh[2], Yusuff Oladosu[1], Md Al Mamun[1], and Atiqullah Khaliqi[2]

[1] Laboratory of Climate-Smart Food Crop Production, Institute of Tropical Agriculture and Food Security (ITAFoS), Universiti Putra Malaysia (UPM), 43400, UPM Serdang, Selangor, Malaysia.
[2]Department of Crop Science, Faculty of Agriculture, Universiti Putra Malaysia (UPM), 43400, UPM Serdang, Selangor, Malaysia.
[3] Bangladesh Agricultural Research Institute (BARI), Gazipur-1701, Bangladesh.

*Correspondence: M. Y. Rafii (mrafii@upm.edu.my), Tel.: +60-3-8947-1042; M.M.H. Khan (mhasan.bari12@gmail.com), Tel.: +60-109187125

Supplementary Materials

Table S1: Mean square and coefficient of variance estimation for quantitative traits revealed by ANOVA.

Trait	Replication (df=2)	Genotype (df=43)	Mean ± SE	Max.	Min.	CV (%)
DTE	2.19	13.65**	8.54±0.20	14.00	4.00	27.09
D50%F	5.55	73.17**	37.91±0.47	52.00	26.00	14.31
DTM	12.19	252.72**	129±0.82	149.00	116.00	7.30
PH	35.8**	11.53**	24.89±0.24	31.37	15.31	11.31
NB	28.00	121.4**	39.14±0.60	59.00	21.00	17.67
NS	224.05**	18.05**	20.87±0.29	30.00	14.00	16.22
NP	1466.89	16996.27**	297.82±6.76	413.00	149.00	26.09
NL	13201.98	152966.44**	893.45±20.29	1239.00	447.00	26.09
NNS	4.46	7.29*	13.13±0.18	25.00	8.00	16.07
IL	0.74*	0.41**	3.32±0.04	5.00	2.40	14.50
BFW	5569.99*	62281.04**	395.46±12.71	650.24	115.82	36.93
BDW	725.43*	12337.99**	239.03±5.61	404.19	147.39	27.01
TNP	103.05*	503.18**	76.08±1.16	101.00	41.00	17.51
NMP	125.55*	491.24**	63.50±1.14	89.00	31.00	20.69
NIP	4.19	15.93**	12.57±0.23	17.00	8.00	21.69
FPW	3358.21**	56883.56**	548.49±11.94	753.05	258.97	25.02
DPW	2940.74**	10852.22**	323.73±5.28	414.25	185.53	18.74
PL	7.96	88.26***	30.85±0.53	44.78	20.08	19.56
PW	0.10	6.50***	14.43±0.17	19.68	9.42	13.18
NSP	109.23*	736.26**	76.88±1.40	106.00	32.00	20.93
DSW	302.80	6671.27**	251.51±4.16	325.48	143.91	19.00
SL	0.50	8.27**	13.72±0.17	20.45	9.83	14.34
SW	1.83	7.13**	10.42±0.15	15.04	6.98	16.84
HSW	398.97	6592.91**	331.59±4.48	491.93	198.92	15.53
SP	78.71*	49.34**	77.88±0.43	86.29	63.69	6.35
HI	18.27*	160.18**	57.69±0.64	70.80	42.72	12.81
Yld	104193.89**	384501.04**	1927.01±31.42	2466.00	1104.00	18.74

Legend: "**" is significant at the 0.01 level; "*" is significant at the 0.05 level, df= degree of freedom, Max = maximum (across genotypes), Min = minimum (across genotypes), CV = coefficient of variation, LSD = least significant difference, Days to emergence = DTE (d), Days to 50% flowering (d) and Days to maturity= DTM (d), Plant height (cm)= PH, Number of branches per plant = NB, Number of stems per plant = NS, Number of petioles per plant = NP, Number of leaves per plant = NL, No. of nodes per stem=NNS, Inter nodes length=IL (cm), Biomass fresh weight per plant=BFW (g) and Biomass dry weight per plant=BDW(g), Total no. of pods per plant = TNP, Number of mature pods per plant = MP, Number of Immature pods per plant = IMP, Fresh pods weight = FPW(g), Dry pods weight = DPW(g), Pod length = PL (mm), Pod width = PW (mm), Number of seeds per plant = NSP, Dry seed weight per Plant = DSW(g), Seed length = SL (mm), Seed width =SW (mm), hundred seed weight = HSW(g), Shelling percent = SP%, Harvest index =HI (%) and Yield = Yld (Kg/ha).

Table S2: Mean performance and comparison of 44 accessions based on 27 phenotypic traits revealed by ANOVA.

Genotypes	DTE	D50%F	DTM	PH	NB	NS	NP	NL	NNS	IL	BFW	BDW	TNP
S3G1	4.67p	37f-j	132ij	26.48a-e	44b-h	16.67o	347.67d-l	1043d-l	12.33e-j	3.53b-g	376.98l-n	246.22de	82.33e-j
S3G2	7k-n	37.67e-i	119o	24.77b-h	36k-q	22.33c-h	277.67pq	833pq	12.33e-j	3.3c-j	333.05n-p	384.71a	70.67lm
S3G3	7k-n	33jk	137e-h	27.62a-c	40.67e-l	19.67h-n	177.67s	533s	11.66g-j	3.33c-i	432.87j-l	207.39l-p	81.33g-j
S3G4	7k-n	37.67e-i	121m-o	23.21d-i	24t	17.33no	302.67m-p	908m-p	12.33e-j	3.03g-k	190.78tu	154.52t	52.33rs
S3G5	4.67p	29kl	120.33	24.98a-h	44b-h	18l-o	315k-p	945k-p	14b-h	4.17a	599.24a-c	222.73g-m	86.67b-g
S3G6	5op	28.67kl	117.67o	26.77a-d	44.33a-h	18l-o	368a-g	1104a-g	17.66a	3.83a-c	554.97c-e	304.78c	91abc
S3G7	5op	28l	120.33no	24.00c-i	47.67a-c	23.33c-f	327g-m	981g-m	12.66d-i	3.27c-k	614.25ab	238.88d-h	88.33a-f
S3G8	5.33n-p	28.67kl	119.67o	26.13a-f	46a-e	21.33e-k	163.33s	490s	14b-h	3.63a-f	513.74e-g	332.28b	93.33a
S3G9	8h-m	34.67g-j	132.33h-j	28.04ab	36.33k-q	18.67k-o	360c-j	1080c-j	11ij	3.53b-g	158.72uv	188.79r-q	85c-g
S3G10	7k-n	37.67e-i	126.67kl	24.55b-i	40f-m	17.33no	225r	675r	11.33h-j	3.73a-e	511.73e-g	201.58n-p	76j-l
S3G11	7k-n	35.33f-j	129.33jk	24.7b-h	37k-q	19.33i-o	308.33l-p	925l-p	12.67d-i	3.13f-k	198.29s-u	157.57st	65.33m-o
S3G12	7.33j-m	38e-i	132.67h-j	22.82e-i	43c-j	20g-n	175.67s	527s	14.33b-g	3.07f-k	495.45f-i	242.2d-g	83.67d-h
S3G13	10c-g	39.67c-f	139.67c-f	22.47f-i	26.67st	16.67o	400.33abc	1201a-c	14.33b-g	3.1f-k	119.37v	214.78i-o	54.67q-s
S3G14	9.67d-h	39.67c-f	119.33o	27.35a-c	34.33n-r	17.67m-o	380a-d	1140a-d	15.33a-d	3.83a-c	389.25k-n	214.42i-o	82.67e-i
S3G15	11.67a-c	38e-i	137.67d-g	25.03a-g	32q-s	19.67h-n	316.67k-p	950k-p	14.33b-g	3.1f-k	619.5a	170.05r-t	88.67a-e
S3G16	10.33b-f	39c-h	120o	24.94a-h	45.67a-e	19.33i-o	279.33op	838op	14.67b-f	3.53b-g	369.07mn	252.86d	71lm
S3G17	9e-j	36f-j	138.33d-f	23.14d-i	35m-r	25a-c	180.33s	541s	12.33e-j	2.97g-k	301.63o-q	378.73a	76.33i-l
S3G18	8.67f-k	36.67f-j	121.67m-o	25.99a-f	35.33l-r	22.33c-h	304.33m-p	913m-p	12.67d-i	3g-k	459.54g-j	203.8m-p	94.33a
S3G19	9.67d-h	41.67b-e	141b-e	21.58g-i	24t	20g-n	323.33h-n	970h-n	16a-c	2.7k	334.67n-p	232.89d-i	55.33q-s
S3G20	9.33d-i	37.33e-j	120.33no	23.35d-i	39.33h-n	19.33i-o	369.67a-e	1109a-e	13.33c-i	3.83a-c	490.4f-i	234.05d-i	58.67p-r
S3G21	10.33b-f	41.67b-e	129jk	25.14a-g	41.33d-k	20.67f-l	329f-m	987f-m	14.67b-f	3.83a-c	449.67h-j	329.39b	78.33h-k
S3G22	10.67a-e	35.67f-j	137.67d-g	22.37f-i	37.67j-p	27a	170.33s	511s	14.67b-f	2.93h-k	280.49p-r	230.81e-j	61o-q
S3G23	12ab	35.67f-j	119o	24.79b-h	45a-g	24b-e	361.67b-i	1085b-i	15a-e	3.3c-j	160.81uv	329.41b	74.33kl
S3G24	9.67d-h	35.33f-j	125k-n	26.41a-e	45.67a-e	21.33e-k	237qr	711qr	13.3c-i	3.2d-k	515.83e-g	229.38e-k	85.67b-g
S3G25	9e-j	34.33ij	138.33d-f	22.92e-i	32.33p-r	20g-n	310l-p	930l-p	12fg-j	3.4c-h	200.39s-u	195.3o-q	71.33lm
S3G26	9.67d-h	38.33d-i	144.33a-c	23.07d-i	39.33h-n	22d-i	182s	546s	11.3h-j	2.8i-k	364.21mn	155.96st	82f-j
S3G27	8.33g-k	46ab	120o	21.18hi	35.33l-r	21.67d-j	402ab	1206ab	14.67b-f	2.73jk	125.8v	242.36d-g	62.33op
S3G28	9.33d-i	43bc	119.67o	20.84i	38.33i-o	19j-o	381.67a-d	1145a-d	16.33ab	2.77i-k	358.01m-o	210.32j-o	88.33a-f
S3G29	11a-d	39.33c-g	122.33l-o	25.72a-f	33.33o-r	19.33i-o	318.33j-p	955j-p	13d-i	3.5b-h	239.52r-t	202.64m-p	58.67p-r
S3G30	9e-j	37f-j	120.67m-o	23.40d-i	30rs	22.33c-h	352d-k	1056d-k	12.66d-i	3.77a-d	368.58mn	176.52r-s	70.33l-n
S3G31	8.67f-k	39.67c-f	117.67o	25.42a-f	49ab	20g-n	282n-p	846n-p	12f-j	3.2d-k	619a	244.02d-f	84d-h
S3G32	9e-j	35.67f-j	133g-j	28.27ab	43c-j	25abc	182s	546s	12.67d-i	3.23d-k	591.39a-d	391.6a	81.67g-j
S3G33	6.67l-o	37f-j	121.67m-o	23.86c-i	44b-h	22.67c-g	307l-p	921l-p	11.67g-j	2.93h-k	539d-f	219.76h-n	92ab
S3G34	6.67l-o	41.67b-e	120.67m-o	25.65a-f	44.67a-h	20.33g-m	319.33j-p	958j-p	13d-i	3.47c-h	504.29e-h	158.41st	85.67b-g
S3G35	7k-n	35.67f-j	136.33e-i	27.42a-c	45.33a-f	22d-i	372.33a-e	1117a-e	13d-i	4.07ab	489.9f-i	225.45f-l	90a-d
S3G36	6.33m-p	42.67b-d	121m-o	24.65b-h	43.33c-i	21f-k	331.33e-m	994e-m	12.33e-j	3.17e-k	559.71b-e	300c	86b-g
S3G37	7.33j-m	34ij	136f-i	26.77a-d	39.67g-n	26.33ab	167.67s	503s	11.67g-j	3.53b-g	515.33e-g	244.97d-f	43.67t
S3G38	7.67i-m	36.67f-j	135f-i	28.69a	49.67a	24.33a-d	364.33a-h	1093a-h	12.67d-i	3.43c-h	426.98j-l	327.49b	82.33e-j
S3G39	7.33j-m	38.33d-i	125.33k-m	25.2a-g	46.67a-d	20g-n	229.33r	688r	13d-i	3.63a-f	279.99p-r	334.25b	71.33lm
S3G40	8.33g-k	35g-j	129.33jk	25.57a-f	38.33i-o	20.33g-m	312.67k-p	938k-p	9.67j	3.03g-k	406.89j-m	204.92l-p	82f-j
S3G41	12.33a	50.33a	143.67a-c	23.46d-i	38i-o	22.33c-h	180s	540s	11ij	2.97g-k	247.63q-s	157.01st	64n-p
S3G42	12.33a	47.67a	147.67a	23.12d-i	40f-m	22d-i	404.67a	1214a	12.33e-j	3g-k	363.71mn	249.41de	82.33e-j
S3G43	11.67a-c	46ab	142.33b-d	28ab	30rs	21.33e-k	384.33a-d	1153a-d	13d-i	3.73a-e	290.84p-r	165.96st	51.33s
S3G44	12ab	48a	144.67ab	25.68 a-f	37k-q	21.33e-k	321i-o	963i-o	13.33c-i	3g-k	439.02i-k	209.15k-p	81.33g-j
Mean	8.54±0.20	37.91±0.47	129.01±0.82	24.90±0.25	39.14±0.60	20.87±0.29	297.82±6.76	893.45±20.29	13.14±0.18	3.32±0.04	395.47±12.71	239.04±5.62	76.08±1.16
Std Dev	2.31	5.42	9.41	2.82	6.92	3.38	77.71	233.13	2.11	0.48	146.06	64.56	13.33
Min	4.67	28.00	117.67	20.84	24.00	16.67	163.33	490.00	9.67	2.70	119.37	154.52	43.67
Max	12.33	50.33	147.67	28.69	49.67	27.00	404.67	1214.00	17.67	4.17	619.50	391.60	94.33

Legend: Number of mature pods per plant = MP, Number of Immature pods per plant = IMP, Fresh pods weight = FPW(g), Dry pods weight = DPW(g), Pod length = PL (mm), Pod width = PW (mm), Number of seeds per plant = NSP, Dry seed weight per Plant = DSW(g), Seed length = SL (mm), Seed width =SW (mm), hundred seed weight = HSW(g), Shelling percent = Shell %, Harvest index =HI (%) and Yield = Yld (Kg/ha). DNMRT = 0.05

Table 2: Continued

Genotypes	NMP	NIP	FPW	DPW	PL	PW	NSP	DSW	SL	SW	HSW	SP	HI	Yld
S3G1	70.67d-i	11.67e-j	674.29b-e	361.21d-g	26.86l-s	13.7d-j	90.33a-c	267.94j-l	12.91g-j	9.11k-q	355.4d-h	74.22m-q	59.45f-h	2150.08d-g
S3G2	55l-n	15.67ab	496.21lm	320.10k-m	26.58l-s	13.95d-j	88a-c	217.62n-q	12.29g-j	8.85l-q	355.55d-h	68.13rs	45.41st	1905.4k-m
S3G3	66.67ij	14.67a-d	639.67fg	379.85a-c	27.42k-r	15.66a-e	95.67a	291.49c-h	12.38g-j	8.43o-q	316.39h-n	76.77f-p	64.69b	2260.99a-c
S3G4	43.67p-r	8.67k	568.87jk	341.39ij	23.02q-s	13.71d-j	90a-c	250.31lm	12.48g-j	8.24pq	369.68a-f	73.42m-r	68.79a	2032.06ij
S3G5	75c-g	11.67e-j	662.11de	369.65c-e	40.76ab	14.21b-j	86cd	278.19f-j	16.29b-d	10.74d-k	339.08e-k	75.26j-q	62.40b-e	2200.3c-e
S3G6	82ab	9jk	635.22g	393.35a	38.89a-c	15.51a-g	91.67a-c	307.37a-c	15.08c-f	13.17ab	355.16d-h	78.26d-m	56.38ij	2341.35a
S3G7	78.67a-c	9.67i-k	680.43b-d	392.43a	38.34a-d	14.97b-h	95ab	311.62ab	14.28d-g	12.55a-c	377.23a-e	79.44b-l	62.16b-e	2335.89a
S3G8	83.67a	9.67i-k	686.92bc	387.77ab	40.3ab	15.56a-f	95.33a	293.45b-g	15.75c-e	13.22ab	341.58e-k	75.68i-q	53.86jk	2308.15ab
S3G9	69g-i	16a	330.15q	204.74r	34.46c-i	14.42b-j	68.67gh	173.02v-x	13.62e-i	9.19j-q	210.79s	84.49ab	52.40k-n	1218.69r
S3G10	60.33k-m	15.67ab	606.01h	360.48e-h	36.11b-g	16.1a-d	60ij	282.49e-j	12.71g-j	8.90l-q	342.84e-k	78.37d-m	64.13b	2145.7e-h
S3G11	56lm	9.33i-k	479.97mn	279.45o	21.63s	12.69h-k	55.67jk	219.28n-p	13.43f-i	8.64m-q	314.66h-n	78.50d-m	63.98b	1663.35o
S3G12	71.67d-i	12d-i	687.17bc	378.54a-c	30.1h-o	16.1a-d	71.33e-h	268.34j-l	13.88e-h	9.18j-q	308.28i-o	70.92q-s	60.977c-f	2253.22a-c
S3G13	39rs	15.67ab	349.37pq	234.85q	26.18m-s	12.49i-k	38.67l	199.32q-t	11.03j	9.5i-q	254.21q-s	84.86a	52.22k-o	1397.9q
S3G14	68hi	14.67a-d	597.42hi	311.95lm	27.38q-r	14.11c-j	67.667hi	253.26kl	11.65ij	9.32j-q	342.51e-k	81.09a-h	59.25f-h	1856.85lm
S3G15	73c-h	15.67ab	657.79ef	377.51a-d	28.88j-p	14.72b-i	72.67e-h	298.13a-e	11.82h-j	8.58n-q	392.13a-d	79.04c-l	68.93a	2247.07a-d
S3G16	57.33lm	13.67a-g	358.56p	253.46p	26.06n-s	13.16f-k	57j	196.04r-t	11.08j	9.35j-q	289.58l-q	77.48e-o	50.07n-q	1508.71p
S3G17	61j-l	15.33a-c	491.54lm	294.64no	33.29d-j	16.41a-c	91a-c	212.63o-r	13.77e-i	10.15f-n	306.68j-o	72.19o-s	43.757t	1753.83no
S3G18	78.33a-c	16a	693b	358.66e-h	31.61g-l	14.45b-j	88.33a-c	284.07e-j	14.38d-g	8.88l-q	333.07f-l	79.23b-l	63.77b	2134.86e-h
S3G19	44.33p-r	11g-k	386.20o	238.74pq	22.25rs	13.17f-k	57.67j	187.98t-v	13.14f-j	9.63i-q	281.21m-r	78.87c-l	50.64l-p	1421.09pq
S3G20	45p-r	13.67a-g	582.11ij	387.41ab	30.45h-n	15b-h	88.33a-c	289.48c-i	13.62e-i	9.79h-p	401.66a-c	74.69k-q	62.34b-e	2306.01ab
S3G21	68.67hi	9.67i-k	558.55k	303.29mn	29.76h-o	15.31a-g	85.33cd	228.70no	13.75e-i	10.14f-n	350.9d-i	75.40i-q	47.97q-s	1805.27mn
S3G22	49.33n-p	11.67e-j	343.76pq	254.49p	30.54h-n	13.10g-k	72e-h	206.56p-t	12.95f-j	10.38e-l	260.42p-r	81.21a-h	52.42k-n	1514.84p
S3G23	59.67k-m	14.67a-d	506.92l	278.49o	29.16i-p	15.02b-h	76.67ef	209.09p-s	13.36f-i	11.05c-i	303.7k-p	75.14j-q	45.83r-t	1657.68o
S3G24	74c-h	11.67e-j	672.81b-e	380.18a-c	32.99d-j	17.55a	90.33a-c	315.68a	13.95e-h	9.48i-q	406.5a	83.04a-d	62.40b-e	2262.99a-c
S3G25	59.67k-m	11.67e-j	389.35o	197.96r	25.35n-s	14.20b-j	59.33j	159.04wx	13.37f-i	7.98q	273.64n-r	80.27a-j	50.32m-q	1178.32r
S3G26	71.67d-i	10.33h-k	669.97c-e	366.30c-e	27.49k-r	12.15j-l	71.33e-h	266.85j-l	13.43f-1	9.95g-o	345.99e-k	72.87n-r	70.12a	2180.38c-e
S3G27	48.33op	14a-f	270.51s	238.92pq	24.96o-s	14.15c-j	48k	192.78s-u	13.61e-i	9.20j-q	269.04o-r	80.63a-i	49.63o-q	1422.19pq
S3G28	75.67c-f	12.67c-h	662.71de	364.23c-f	32.71e-k	15.62a-e	75.33e-h	286.06d-j	12.03h-j	9.60i-q	362.02b-g	78.59d-m	63.39bc	2168.04c-f
S3G29	46pq	12.67c-h	484.09mn	294.14no	27.91j-q	13.57eij	87.33bc	231.85mn	12.99f-j	10.80d-j	327.57f-l	78.72d-m	59.23f-h	1750.83no
S3G30	56.33lm	14a-f	402.79o	197.94r	26.41l-s	14.19b-j	78.33de	153.81x	13.82e-h	11.46c-h	265.91o-r	77.88d-n	52.83k-m	1178.19r
S3G31	70e-i	14a-f	593.62hi	334.76i-k	34.79c-h	14.91b-i	85.67cd	253.99kl	13.89e-h	11.79b-f	323.32g-m	75.93h-q	57.83hi	1992.62i-k
S3G32	69.67f-i	12d-i	659.54ef	364.15c-f	29.69h-o	15.49a-g	88a-c	295.26b-g	13.51f-i	10.86c-j	379.56a-e	81.16a-h	48.187p-r	2167.55c-f
S3G33	78a-c	14a-f	627.47g	372.76b-e	41.30ab	14.86b-i	91a-c	285.05d-j	18.95a	11.94b-e	360.39c-g	76.60g-p	62.9b-d	2218.84b-e
S3G34	76b-e	9.67i-k	598.54hi	346.74g-i	37.28a-f	15.92a-e	90.67a-c	271.02i-k	16.97a-c	12.42a-d	378.89a-e	78.13d-n	68.65a	2063.9g-i
S3G35	75c-g	15a-c	658.94ef	348.52f-i	42.18a	15.08b-h	90a-c	273.80h-j	16.97a-c	14.03a	327.05f-l	78.77d-l	60.66d-g	2074.54f-i
S3G36	76.33b-d	9.67i-k	740.11a	337.18ij	37.82a-e	14.38b-j	89.33a-c	276.27g-j	18.05ab	13.91a	330.94f-l	81.99a-f	52.90k-m	2007ij
S3G37	33.33s	10.33h-k	293.63r	242.21pq	30.27h-o	13.51e-j	33l	174.43u-w	12.48g-j	10.46e-l	245.22rs	72.14p-s	49.70o-q	1441.75pq
S3G38	71d-i	11.33f-k	689.37bc	371.35b-e	31.56g-m	15.76a-e	70.67e-h	295.52b-e	13.95e-h	11.98b-e	358.03c-h	79.58a-k	53.147kl	2210.45b-e
S3G39	57lm	14.33a-e	469.32n	328.41j-l	32.39f-k	16.63ab	56.67j	221.93n-p	13.75e-i	10.76d-k	306.46j-o	67.50s	49.55pq	1954.82j-l
S3G40	69.33g-i	12.67c-h	591.34hi	343.69h-j	36.04b-g	14.94b-h	69f-h	289.45c-i	12.95f-j	11.56b-g	348.03e-j	84.17a-c	62.61b-e	2045.77h-j
S3G41	54.33m-o	9.67i-k	282.61rs	372.46b-e	24.89o-s	9.90l	76.33e-g	304.08a-d	13.04f-j	10.69e-k	378.31a-e	81.7a-g	70.34a	2217.02b-e
S3G42	69.33g-i	13b-h	672.03c-e	373.79b-e	28.03j-q	15.223a-g	87.33bc	305.59a-c	14.28d-g	11.93b-e	405.2ab	81.80a-g	59.97e-h	2224.91b-e
S3G43	41.67qr	9.67i-k	363.71p	228.82q	24.11p-s	11.03kl	75e-h	188.36t-v	12.71g-j	10.32e-m	290.49l-q	82.35a-e	58.01g-i	1362.01q
S3G44	65.67i-k	15.67ab	668.77c-e	377.51a-d	29.65h-o	12.65h-k	87c	303.55a-d	13.43f-i	10.63e-k	404.78ab	80.46a-j	64.403b	2247.07a-d
Mean	63.50±1.14	12.57±0.24	548.48±11.94	323.73±5.28	30.85±0.53	14.43±0.17	76.87±1.40	251.60±4.16	13.72±0.17	10.42±0.15	331.59±4.48	77.88±0.43	57.69±0.64	1927.01±31.42
Std Dev	13.14	2.73	137.23	60.65	6.04	1.90	16.09	47.82	1.97	1.76	51.49	4.94	7.39	361.03
Min	33.33	8.67	270.51	197.94	21.63	9.90	33.00	153.81	11.03	7.98	210.79	67.50	43.76	1178.19
Max	83.67	16.00	740.11	393.35	42.18	17.55	95.67	315.68	18.95	14.03	406.50	84.86	70.34	2341.35

Legend: Number of mature pods per plant = MP, Number of Immature pods per plant = IMP, Fresh pods weight = FPW(g), Dry pods weight = DPW(g), Pod length = PL (mm), Pod width = PW (mm), Number of seeds per plant = NSP, Dry seed weight per Plant = DSW(g), Seed length = SL (mm), Seed width =SW (mm), hundred seed weight = HSW(g), Shelling percent = SP, Harvest index =HI (%) and Yield = Yld (Kg/ha).

Table S3: Estimation of Pearson's correlation matrix for 27 quantitative traits of 44 Bambara groundnut accessions.

Traits	DTE	D50%F	DTM	PH	NB	NS	NP	NL	NdS	IL	BFW	BDW	TNP	NMP
DTE	1	0.521**	0.413**	-0.1	-0.307*	0.156	0.068	0.068	0.019	-0.252*	-0.332**	-0.153	-0.319*	-0.363**
D50%F		1	0.296*	-0.194*	-0.250*	0.001	0.208*	0.208*	-0.009	-0.266*	-0.270*	-0.196*	-0.239*	-0.254*
DTM			1	-0.031	-0.283*	0.162	-0.192*	-0.192*	-0.182*	-0.218*	-0.197*	-0.183*	-0.228*	-0.228*
PH				1	0.245*	-0.048	0.007	0.007	0.027	0.364**	0.202*	0.114	0.171*	0.156
NB					1	0.16	-0.158	-0.158	-0.017	0.224*	0.480**	0.364**	0.528**	0.550**
NS						1	-0.282*	-0.282*	-0.142	-0.098	0.03	0.254*	0.008	0.017
NP							1	1.00**	0.234*	0.143	-0.195*	-0.159	0.022	-0.003
NL								1	0.234*	0.143	-0.195*	-0.159	0.022	-0.003
NdS									1	0.045	0.021	0.131	0.02	0.027
IL										1	0.182*	0.063	0.086	0.069
BFW											1	0.153	0.523**	0.551**
BDW												1	0.156	0.146
TNP													1	0.978**
NMP														1

	NIP	FPW	DPW	PL	PW	NSP	DSW	SL	SW	HSW	SP	HI	Yld
DTE	0.190*	-0.315*	-0.235*	-0.475**	-0.273*	-0.187*	-0.151	-0.363**	-0.181*	0.028	0.233*	-0.019	-0.235*
D50%F	0.054	-0.214*	-0.11	-0.377**	-0.279*	-0.151	-0.047	-0.126	-0.084	0.133	0.178*	0.104	-0.11
DTM	-0.012	-0.183*	-0.118	-0.294*	-0.277*	-0.227*	-0.058	-0.197*	-0.126	-0.099	0.190*	0.076	-0.118
PH	0.086	0.166	0.095	0.132	0.051	0.16	0.114	0.077	0.112	0.038	0.045	-0.019	0.095
NB	-0.067	0.429**	0.481**	0.504**	0.337**	0.282*	0.434**	0.390**	0.444**	0.289*	-0.166*	-0.002	0.481**
NS	-0.039	-0.074	0.004	0.033	0.011	0.067	-0.047	0.101	0.278*	-0.096	-0.184*	-0.207*	0.004
NP	0.127	0.009	-0.183*	-0.016	-0.072	-0.011	-0.093	0.089	0.075	0.023	0.290*	-0.014	-0.183*
NL	0.127	0.009	-0.183*	-0.016	-0.072	-0.011	-0.093	0.089	0.075	0.023	0.290*	-0.014	-0.183*
NdS	-0.027	-0.003	-0.046	-0.03	0.078	-0.072	-0.045	-0.047	-0.042	-0.029	0.003	-0.156	-0.046
IL	0.086	0.062	0.001	0.265*	0.11	0.135	-0.048	0.124	0.147	-0.038	-0.142	-0.057	0.001
BFW	-0.1	0.651**	0.611**	0.536**	0.321*	0.414**	0.599**	0.360**	0.356**	0.453**	-0.067	0.256*	0.611**
BDW	0.059	0.154	0.115	0.205*	0.356**	0.176*	0.029	0.084	0.229*	0.046	-0.289*	-0.740**	0.115
TNP	0.170*	0.732**	0.584**	0.592**	0.393**	0.534**	0.594**	0.394**	0.330*	0.379**	0.037	0.241*	0.584**
NMP	-0.034	0.751**	0.611**	0.588**	0.380**	0.564**	0.623**	0.430**	0.366**	0.401**	0.04	0.270*	0.611**
NIP	1	-0.039	-0.089	0.062	0.091	-0.105	-0.099	-0.147	-0.152	-0.084	-0.011	-0.125	-0.089
FPW		1	0.815**	0.454**	0.389**	0.629**	0.784**	0.344**	0.277*	0.638**	-0.1	0.411**	0.815**
DPW			1	0.399**	0.285*	0.581**	0.943**	0.273*	0.261*	0.752**	-0.179*	0.569**	1.00**
PL				1	0.399**	0.377**	0.406**	0.555**	0.543**	0.163	0.016	0.071	0.399**
PW					1	0.238*	0.240*	0.207*	0.131	0.134	-0.143	-0.126	0.285*
NSP						1	0.559**	0.390**	0.314*	0.547**	-0.076	0.255*	0.581**
DSW							1	0.264*	0.277*	0.782**	0.15	0.598**	0.943**
SL								1	0.591**	0.183*	-0.03	0.104	0.273*
SW									1	0.180*	0.025	-0.028	0.261*
HSW										1	0.045	0.482**	0.752**
SP											1	0.103	-0.179*
HI												1	0.569**
Yld													1

Legend: "**" is significant at the 0.01 level; "*" is significant at the 0.05 level, Days to emergence = DTE (d), Days to 50% flowering (d) and Days to maturity= DTM (d), Plant height (cm)= PH, Number of branches per plant = NB, Number of stems per plant = NS, Number of petioles per plant = NP, Number of leaves per plant = NL, No. of nodes per stem=NNS, Inter nodes length=IL (cm), Biomass fresh weight per plant=BFW (g) and Biomass dry weight per plant=BDW(g), Total no. of pods per plant = TNP, Number of mature pods per plant = MP, Number of Immature pods per plant = IMP, Fresh pods weight = FPW(g), Dry pods weight = DPW(g), Pod length = PL (mm), Pod width = PW (mm), Number of seeds per plant = NSP, Dry seed weight per Plant = DSW(g), Seed length = SL (mm), Seed width =SW (mm), hundred seed weight = HSW(g), Shelling percent = SP, Harvest index =HI (%) and Yield = Yld (Kg/ha).

Table S4: Estimation of genetic parameters 27 quantitative traits of 44 Bambara groundnut accessions.

Traits	Mean	σ_e^2	σ_g^2	σ_p^2	PCV (%)	GCV (%)	RD (%)	h_b^2 %	GA (%)
**DTE**	8.54	1.27	4.12	5.40	27.21	23.79	12.60	76.39	42.82
**D50%F**	37.91	8.09	21.69	29.78	14.40	12.29	14.65	72.84	21.60
**DTM**	129.01	8.27	81.48	89.76	7.34	7.00	4.72	90.78	13.73
**PH**	24.90	5.48	2.02	7.50	11.00	5.70	48.15	26.88	6.09
**NB**	39.14	11.49	36.64	48.13	17.72	15.46	12.75	76.13	27.79
**NS**	20.87	3.22	4.94	8.16	13.69	10.65	22.16	60.59	17.08
**NP**	297.82	666.24	5443.30	6109.54	26.25	24.77	5.61	89.10	48.17
**NL**	893.45	5996.10	48990.10	54986.20	26.25	24.77	5.61	89.10	48.17
**NNS**	13.14	3.04	1.42	4.46	16.07	9.05	43.64	31.76	10.51
**IL**	3.32	0.13	0.09	0.22	14.25	9.22	35.32	41.83	12.28
**BFW**	395.47	1225.00	20352.00	21577.00	37.14	36.07	2.88	94.32	72.17
**BDW**	239.04	163.69	4058.10	4221.79	27.18	26.65	1.96	96.12	53.82
**TNP**	76.08	16.48	162.23	178.71	17.57	16.74	4.72	90.78	32.86
**NMP**	63.51	14.37	158.96	173.33	20.73	19.85	4.24	91.71	39.16
**NIP**	12.58	3.27	4.22	7.49	21.76	16.34	24.92	56.37	25.27
**FPW**	548.49	164.37	18906.40	19070.77	25.18	25.07	0.43	99.14	51.42
**DPW**	323.74	109.31	3581.00	3690.31	18.76	18.48	1.49	97.04	37.51
**PL**	30.86	11.19	25.69	36.89	19.68	16.42	16.54	69.65	28.24
**PW**	14.43	2.26	1.41	3.67	13.28	8.23	38.03	38.40	10.51
**NSP**	76.88	23.72	237.51	261.24	21.02	20.05	4.65	90.92	39.38
**DSW**	251.61	140.11	2177.10	2317.21	19.13	18.54	3.07	93.95	37.03
**SL**	13.72	1.76	2.17	3.93	14.44	10.74	25.65	55.28	16.45
**SW**	10.43	1.09	2.01	3.10	16.89	13.61	19.44	64.90	22.59
**HSW**	331.59	733.20	1953.20	2686.40	15.63	13.33	14.73	72.71	23.41
**SP**	77.89	10.74	12.87	23.60	6.24	4.61	26.17	54.51	7.01
**HI**	57.70	2.68	52.50	55.18	12.87	12.56	2.46	95.15	25.23
**Yld**	1927.01	3873.00	126876.00	130749.00	18.76	18.48	1.49	97.04	37.51

Legend: σ_e^2 = Error variance; σ_g^2 = Genotypic variance; σ_p^2 = Phenotypic variance; h_b^2 = Heritability in broad sense; PCV = Phenotypic coefficient of variation; GCV = Genotypic coefficient of variation; RD = Relative difference; GA = Genetic advance. Days to emergence = DTE (d), Days to 50% flowering (d) and Days to maturity= DTM (d), Plant height (cm)= PH, Number of branches per plant = NB, Number of stems per plant = NS, Number of petioles per plant = NP, Number of leaves per plant = NL, No. of nodes per stem=NNS, Inter nodes length=IL (cm), Biomass fresh weight per plant=BFW (g) and Biomass dry weight per plant=BDW(g), Total no. of pods per plant = TNP, Number of mature pods per plant = MP, Number of Immature pods per plant = IMP, Fresh pods weight = FPW(g), Dry pods weight = DPW(g), Pod length = PL (mm), Pod width = PW (mm), Number of seeds per plant = NSP, Dry seed weight per Plant = DSW(g), Seed length = SL (mm), Seed width =SW (mm), hundred seed weight = HSW(g), Shelling percent = SP, Harvest index =HI (%) and Yield = Yld (Kg/ha).

Table S5: Estimation of Shannon's diversity index (H') and principal component analysis (PCA) of 44 Bambara groundnut accessions.

Parameters	PC1	PC2	PC3	PC4	PC5	PC6	PC7
Eigenvalue	8.88	3.49	2.86	1.92	1.78	1.45	1.27
Proportion of Variance (%)	32.91	12.91	10.60	7.10	6.60	5.38	4.69
Cumulative Variance (%)	32.91	45.82	56.41	63.51	70.10	75.48	80.17
DTE	-0.16	0.23	-0.05	0.29	0.26	0.19	0.16
D50%F	-0.12	0.30	0.02	0.28	0.18	-0.06	0.10
DTM	-0.11	0.20	-0.23	0.27	-0.08	0.24	0.07
PH	0.12	-0.12	0.05	0.04	-0.25	0.46	0.46
NB	0.24	-0.18	-0.05	0.10	0.00	0.04	0.09
NS	0.00	-0.16	-0.30	0.47	0.08	-0.03	0.01
NP	-0.04	0.09	0.54	0.09	0.09	-0.03	0.06
NL	-0.04	0.09	0.54	0.09	0.09	-0.03	0.06
NNS	-0.01	-0.06	0.23	-0.09	0.37	-0.30	0.14
IL	0.07	-0.23	0.22	-0.17	-0.24	0.16	0.45
BFW	0.26	-0.03	-0.05	0.00	-0.07	0.02	0.09
BDW	0.09	-0.33	-0.10	0.12	0.42	-0.01	0.22
TNP	0.27	0.00	0.10	0.05	0.08	0.21	-0.28
NMP	0.28	0.01	0.09	0.06	0.03	0.11	-0.23
NIP	-0.04	-0.02	0.08	-0.06	0.31	0.57	-0.26
FPW	0.29	0.12	0.03	-0.07	0.11	0.05	0.00
DPW	0.29	0.20	-0.11	-0.09	0.09	-0.04	0.10
PL	0.26	-0.18	0.09	0.12	-0.14	0.02	-0.22
PW	0.19	-0.20	0.03	-0.12	0.27	0.14	-0.17
NSP	0.24	0.07	0.01	0.07	0.05	-0.05	0.11
DSW	0.28	0.25	-0.06	0.01	0.05	0.02	0.08
SL	0.20	-0.11	0.10	0.28	-0.20	-0.24	-0.22
SW	0.18	-0.14	0.11	0.44	-0.14	-0.21	0.09
HSW	0.23	0.29	-0.04	-0.01	0.17	-0.08	0.25
SP	-0.04	0.19	0.24	0.34	-0.16	0.22	-0.09
HI	0.12	0.42	-0.01	-0.16	-0.29	-0.03	-0.10
Yld	0.29	0.20	-0.11	-0.09	0.09	-0.04	0.10

Legend: Days to emergence = DTE (d), Days to 50% flowering (d) and Days to maturity= DTM (d), Plant height (cm)= PH, Number of branches per plant = NB, Number of stems per plant = NS, Number of petioles per plant = NP, Number of leaves per plant = NL, No. of nodes per stem=NNS, Inter nodes length=IL (cm), Biomass fresh weight per plant=BFW (g) and Biomass dry weight per plant=BDW(g), Total no. of pods per plant = TNP, Number of mature pods per plant = MP, Number of Immature pods per plant = IMP, Fresh pods weight = FPW(g), Dry pods weight = DPW(g), Pod length = PL (mm), Pod width = PW (mm), Number of seeds per plant = NSP, Dry seed weight per Plant = DSW(g), Seed length = SL (mm), Seed width =SW (mm), hundred seed weight = HSW(g), Shelling percent = SP, Harvest index =HI (%) and Yield = Yld (Kg/ha).
